# NIR‐II Excitation Phototheranostic Platform for Synergistic Photothermal Therapy/Chemotherapy/Chemodynamic Therapy of Breast Cancer Bone Metastases

**DOI:** 10.1002/advs.202204718

**Published:** 2022-10-10

**Authors:** Pengfei Sun, Fan Qu, Chi Zhang, Pengfei Cheng, Xiangyu Li, Qingming Shen, Daifeng Li, Quli Fan

**Affiliations:** ^1^ State Key Laboratory of Organic Electronics and Information Displays & Institute of Advanced Materials Jiangsu Key Laboratory for Biosensors Nanjing University of Posts & Telecommunications Nanjing 210023 China; ^2^ Department of Orthopedics The First Affiliated Hospital of Zhengzhou University Zhengzhou 450052 China

**Keywords:** breast cancer bone metastases, donor–acceptor–donor small molecule, near‐infrared‐II excitation, near‐infrared‐II photothermal therapy, phototheranostic

## Abstract

To improve bone metastases treatment efficacy, current strategies are focused on the integration of chemotherapy with phototheranostic. However, the success of phototheranostic approaches is hampered by the limited tissue penetration depth of near‐infrared‐I (NIR‐I) light (700–900 nm). In this study, a NIR‐II (1000–1700 nm) excitation phototheranostic (BTZ/Fe^2+^@BTF/ALD) is presented for NIR‐II fluorescence imaging and NIR‐II photoacoustic imaging‐guided NIR‐II photothermal therapy (PTT), chemotherapy, and chemodynamic therapy (CDT) of breast cancer bone metastases. This phototheranostic is developed by integrating a dopamine‐modified NIR‐II absorbing donor–acceptor–donor small molecule (BBT‐FT‐DA), the boronate anticancer drug bortezomib (BTZ), and Fe^2+^ ions, as CDT catalysts, into an amphiphilic PEGylated phospholipid modified with the bone‐targeting ligand alendronate. In acidic and hydrogen peroxide (H_2_O_2_) over expression tumor microenvironment, the boronate–catechol linkage is cleaved and BTZ and Fe^2+^ ions are released to initiate the Fenton reaction, that is, chemotherapy and CDT, respectively, are initialized. It is confirmed using the murine 4T1 bone metastasis model that BTZ/Fe^2+^@BTF/ALD significantly suppresses the progression of tumor cells in the bone tissue via a synergistic NIR‐II PTT/chemotherapy/CDT effect. Overall, this work provides fresh insights to guide the development of NIR‐II phototheranostics for breast cancer bone metastases.

## Graphical abstract

A near‐infrared‐II (NIR‐II) (1000–1700 nm) excitation phototheranostic (BTZ/Fe^2+^@BTF/ALD) is developed by integrating a dopamine‐modified NIR‐II absorbing small molecule, the anticancer drug bortezomib, and Fe^2+^ ions, into bone‐targeting ligand alendronate modified amphiphilic PEGylated phospholipid for NIR‐II fluorescence imaging and NIR‐II photoacoustic imaging‐guided NIR‐II photothermal, chemotherapy, and chemodynamic therapy of breast cancer bone metastases.

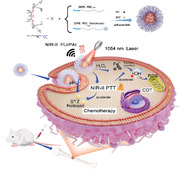

## 1 Introduction

Breast cancer is the most frequently occurring cancer among women worldwide. More than half of advanced‐stage breast cancer patients will have bone metastases, in which cancer cells spread from the primary tumor to distant bones.^[^ ^1^ ^]^ Bone metastasis is commonly associated with skeletal‐related events—such as hypercalcemia, fracture, and spinal cord compression—which seriously decrease patient quality of life and reduce survival expectancy.^[^ ^2^ ^]^ Chemotherapy is the main approach used to inhibit breast cancer growth, reduce the occurrence of skeletal‐related events, and improve patient quality of life.^[^ ^3^ ^]^ However, such treatments have failed to improve the clinical outcomes of breast cancer patients because of their significant side effects including drug resistance, non‐specific biodistributions, and clinical complications.^[^ ^4^ ^]^ Therefore, the development of innovative and effective anti‐tumor therapeutic methods would greatly benefit the clinical treatment of breast cancer bone metastases.^[^ ^6^ ^]^

Phototheranostic strategies, as preeminent protocols for cancer and cancer metastasis treatment, have attracted increasing attention in recent years, mainly because of the advantages of high spatiotemporal resolution and the precise controllability of the light used.^[^ ^7^, ^8^ ^]^ Phototheranostic strategies involve photo‐active agents converting photonic energy into optical signals (detected, for example, via fluorescence imaging [FI] or photoacoustic imaging [PAI]) and therapeutic effects (including photothermal therapy [PTT] and photodynamic therapy [PDT]),^[^ ^9^ ^]^ providing real‐time diagnosis and concurrent in situ therapy.^[^ ^10^ ^]^ However, due to acquired heat shock effects and the hypoxic nature of the tumor microenvironment, PTT and PDT suffer from poor therapeutic efficacy.^[^ ^11^ ^]^ Therefore, in phototheranostics, the current trend is to combine PTT and/or PDT with other therapeutic modalities to mitigate these problems.^[^ ^12^ ^]^ For instance, multifunctional phototheranostic nanoagents have been reported with chemotherapeutic, chemodynamic therapeutic,^[^ ^13^ ^]^ and immunotherapeutic effects.^[^ ^14^, ^15^, ^16^ ^]^ Among versatile multifunctional phototheranostic approaches, PTT associated with chemodynamic therapy (CDT) or chemotherapy has received the greatest attention because the photothermal effect can be utilized to enhance drug release and the Fenton reaction rate in a synergistic effect.^[^ ^17^ ^]^ More importantly, CDT mediated by the Fenton reaction, involving a catalytic decomposition of tumor endogenous H_2_O_2_ to generate the cytotoxic hydroxyl radical (·OH),^[^ ^18^ ^]^ effectively induces tumor cell apoptosis regardless of the degree of hypoxia of the microenvironment of deep tumors.^[^ ^19^, ^20^ ^]^ Although such PTT/CDT or PTT/chemotherapy phototheranostic systems have been shown to result in improved therapeutic outcomes,^[^ ^21^ ^]^ limited tissue penetration depths have been demonstrated for commonly used nanoagents because excitation was confined to the near‐infrared‐I window (NIR‐I, 700–900 nm), and this light is strongly scattered in living tissue.^[^ ^22^, ^23^ ^]^

Compared to NIR‐I light, NIR‐II light (1000–1700 nm) possesses the advantages of less scattering, tissue penetration, and higher maximum permissible exposure (1 W cm^−2^ for 1064 nm and 0.33 W cm^−2^ for 808 nm).^[^ ^24^, ^25^ ^]^ Therefore, NIR‐II PAI, NIR‐II FI, and NIR‐II PTT should provide higher imaging contrast, resolution, and penetration depth, as well as superior photothermal‐conversion efficiency.^[^ ^26^ ^]^ A variety of NIR‐II absorption or NIR‐II fluorescence agents, such as gold nanomaterials, copper sulfide nanomaterials, and conjugated polymers (CPs), have been developed as NIR‐II excitation multifunctional phototheranostic agents.^[^ ^27^ ^]^ In comparison with metal nanomaterials, organic CPs possess the salient advantages of outstanding biocompatibility and ease of processability,^[^ ^28^ ^]^ and hence, they have generated tremendous interest among NIR‐II PTT/chemotherapy, NIR‐II PTT/gas therapy, and NIR‐II PTT/CDT researchers.^[^ ^29^ ^]^ Nevertheless, there are two major challenges for CP‐based phototheranostics that compromise their clinical applicability.^[^ ^30^, ^31^ ^]^ First, CP molecular weights are usually >8000 Da, and the inherently nondegradable nature of large *π*‐conjugated molecular structures means that they possess long total body clearance half‐lives.^[^ ^32^, ^33^ ^]^ Second, CPs are strongly hydrophobic and have fewer functional groups available for the conjugation of drug molecules and iron ions.^[^ ^34^ ^]^ Preparation methods for drug‐loaded CP nanoagents are mainly based on physical encapsulation, which can result in low drug loading efficacies, instability, and burst drug release.^[^ ^35^, ^36^ ^]^ Therefore, the development of NIR‐II‐absorbing small molecules for NIR‐II FI and NIR‐II PTT that have functional group modifications to create effective drug or iron carrier groups is an unmet need.

**Scheme 1:**
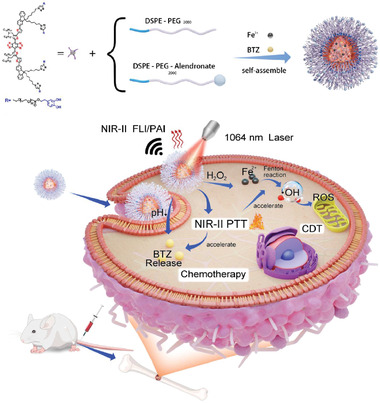
Schematic illustration of 1064 nm light‐activated NIR‐II phototheranostic nanoplatform (BTZ/Fe^2+^@BTF/ALD) for combined NIR‐II PTT/chemotherapy/CDT of breast cancer bone metastases.

As an emerging category of organic photonic materials, small molecules with donor–acceptor–donor (D–A–D) structures have gained increasing attention by virtue of their tunable photophysical properties, good biocompatibility, and chemical flexibility. Currently, D–A–D small molecules have been extensively exploited for NIR‐II FI and NIR‐I PTT. Herein, we reported a NIR‐II excitation multifunctional phototheranostic platform for NIR‐II FI/NIR‐II PAI guided synergistic NIR‐II PTT/chemotherapy/CDT. Dopamine (DA) modified D–A–D small molecules (BBT‐FT‐DA) were synthesized to serve both as the NIR‐II FI/NIR‐II PAI/NIR‐II PTT photoactive agent and drug carrier (**Scheme** **1**). BBT‐FT‐DA was then used to prepare a multifunctional phototheranostic agent (BTZ/Fe^2+^@BTF/ALD) in which the anticancer drug bortezomib (BTZ) and a CDT catalyst (Fe^2+^ ions) were encapsulated within a DSPE‐mPEG2000 shell that is decorated with dopamine groups, which have a high binding affinity toward BTZ and Fe^2+^ ions. The bone targeting ligand alendronate (ALD) was used to modify the surface of BTZ/Fe^2+^@BTF surface and endow it with the capacity to precisely diagnose breast cancer bone metastases and phototheranostic capabilities. Once this phototheranostic agent reaches the breast cancer and bone metastases sites, the acidity and high H_2_O_2_ concentration of the tumor microenvironment will degrade the borate–ester bond, promote the release of the anticancer drug BTZ from BTZ/Fe^2+^@BTF/ALD, generate the cytotoxic hydroxyl radical (·OH), and accelerate the decomposition of the nanoparticles. Upon NIR‐II light irradiation, BTZ/Fe^2+^@BTF/ALD not only allows diagnosis via NIR‐II FI and NIR‐II PAI of breast cancer bone metastases but also triggers the NIR‐II PTT effect. More importantly, the NIR‐II photothermal transduction will enhance the BTZ release rate and Fenton reaction rate, and thus, a synergistic effect in breast cancer treatment will be achieved. Overall, the as‐prepared BTZ/Fe^2+^@BTF/ALD multifunctional phototheranostic platform may provide a seamless synergy for specific imaging and therapy of breast cancer bone metastases.

## 2 Results and Discussion

### 2.1 Preparation and Characterization of BTZ/Fe^2+^@BTF‐DA

**Scheme 2:**
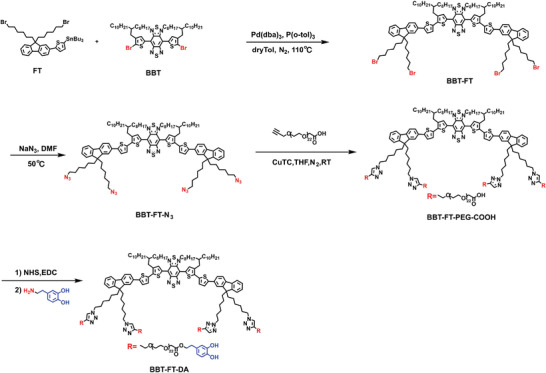
Synthesis route of small organic molecule BBT‐FT‐DA.

The synthetic route of BBT‐FT‐DA is illustrated in **Scheme** **2**. The D–A–D^[^ ^37^, ^38^ ^]^ NIR‐II FL molecule BBT‐FT was synthesized via Stille coupling between BBT and FT. In the D–A–D type molecule BBT‐FT, BBT, which has a strong electron‐withdrawing ability, acts as an acceptor to narrow the band gap,^[^ ^5^ ^]^ thiophene is a strong electron donor and red‐shifts the absorption spectrum to 1064 nm, and 9,9′‐bis(bromohexyl)‐fluorene (F‐Br), is a modifiable moiety selected to allow side chain post‐functionalization. BBT‐FT was converted to BBT‐FT‐N_3_ via substitution of bromide with NaN_3_, and then grafted with carboxylic acid modified PEG1000‐COOH via a click reaction to obtain BBT‐FT‐PEG‐COOH.^[^ ^39^ ^]^ Finally, BBT‐TF‐DA was synthesized by means of an amidation reaction between amino dopamine and BBT‐FT‐PEG‐COOH (Scheme 2). The structures of BBT‐FT, BBT‐FT‐N_3_, and BBT‐FT‐DA were characterized by ^1^H NMR (Figures S1–S3, Supporting Information). In addition, the absorption and fluorescence spectra of BBT‐FT‐DA in THF were recorded, and a strong absorption at 1064 nm and a fluorescence emission band centered at 1124 nm were observed (Figure S4, Supporting Information).

**Figure 1:**
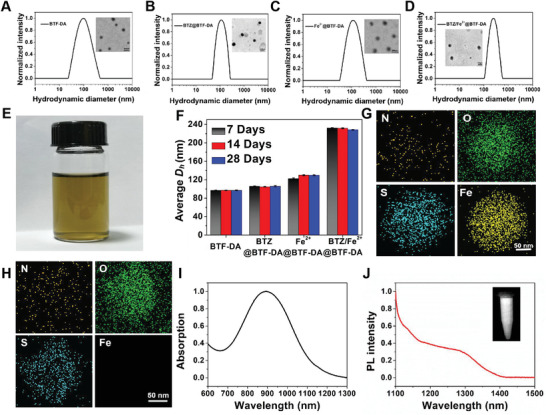
DLS results and TEM images (insets) of A) BTF‐DA, B) BTZ@BTF‐DA, C) Fe^2+^@BTF‐DA, and D) BTZ/Fe^2+^@BTF‐DA in aqueous solution (scale bars: 200 nm). E) Photograph of BTZ/Fe^2+^@BTF‐DA solution. F) Average diameters of BTF‐DA, BTZ@BTF‐DA, Fe^2+^@BTF‐DA, and BTZ/Fe^2+^@BTF‐DA in water after 7, 14, and 28 days of storage. Error bars, mean ± SD (*n* = 3). TEM‐EDS element mapping of G) BTZ/Fe^2+^@BTF‐DA (scale bar: 50 nm), H) BTF‐DA (scale bar: 50 nm). I) Absorption and J) fluorescence emission spectra of BTZ/Fe^2+^@BTF‐DA (0.1 mg mL^−1^) in aqueous solution (Inset: NIR‐II fluorescence images of BTZ/Fe^2+^@BTF‐DA aqueous solution acquired under irradiation at 1064 nm).

The dopamine groups^[^ ^40^ ^]^ in the side chain of BBT‐FT‐DA endow the molecule with efficient conjugation affinity for BTZ and Fe^2+^ ions,^[^ ^16^ ^]^ via highly reactive boronic acid–diol conjugation and coordination interactions, respectively. NIR‐II fluorescence monomer BBT‐FT‐DA, anticancer drug BTZ, and Fe^2+^ ions were incorporated into DSPE‐mPEG2000 to form BTF‐DA/BTZ/Fe^2+^ nanoparticles (BTZ/Fe^2+^@BTF‐DA). To avoid the instability of Fe^2+^ ions that are easily oxidized, the prepared process of BTZ/Fe^2+^@BTF‐DA were following next step: 1) The BTZ@BTF‐DA nanoparticles were first prepared through the bottom‐up method by simply mixing BTZ and BTF‐TF‐DA solutions with DSPE‐PEG2000 under sonicated, 2) and then the top‐down method was used to prepare the BTZ/Fe^2+^@BTF‐DA via coordination interactions between dopamine groups and Fe^2+^ ions by stirring ferrous sulfate solution with BTZ/BTF‐DA nanoparticles under vigorously overnight. To make a meaningful comparison, three nanoparticles only loaded with BBT‐FT‐DA, BTZ, or Fe^2+^ ions (BTF‐DA, BTZ@BTF‐DA, and Fe^2+^@BTF‐DA) were also prepared as a control. **Figure** **1**A–D shows the hydrodynamic diameter *D*_h_ results for BTZ/Fe^2+^@BTF‐DA and the other three nanoparticle types, as measured by dynamic light scattering (DLS). At 246 nm, *D*_h_ for the BTZ/Fe^2+^@BTF‐DA nanoparticles was larger than those for the BTF‐DA, BTZ@BTF‐DA, and Fe^2+^@BTF‐DA nanoparticles. The transmission electron microscopy (TEM) results also indicated a larger size for BTZ/Fe^2+^@BTF‐DA (240 nm) relative to those of BTF‐DA (90 nm), BTZ@BTF‐DA (100 nm), and Fe^2+^@BTF‐DA (110 nm) (Figure 1A–D). Furthermore, the TEM images show that the nanoparticles all possess homogenous sphere‐like morphologies. This value of *D*_h_ was basically unchanged for BTZ/Fe^2+^@BTF‐DA after 4 weeks of storage in the dark at 4 °C (Figure 1F), implying that these nanoparticles have good colloidal stability in vitro. To image the iron distribution in the BTZ/Fe^2+^@BTF‐DA nanoparticles, transmission electron microscopy‐energy dispersive X‐ray spectroscopy (TEM‐EDS) was performed (Figure 1G). Like the O and S elements, Fe was found to be distributed evenly across the entire structure of the BTZ/Fe^2+^@BTF‐DA nanoparticle structure. In addition, no Fe was found in BTZ/Fe^2+^@BTF‐DA, as shown in Figure 1H.

The UV–vis‐NIR absorption spectra of BTZ/Fe^2+^@BTF‐DA were found to have an intense band in the 900–1200 nm range (Figure 1I), and the extinction coefficient at 1064 nm was 1.037 × 10^4^ M^−1^ cm^−1^, which is similar to the 1064 nm extinction coefficients of previously reported NIR‐II PTT nanoagents (Figures S5 and S6, Supporting Information). Meanwhile, a strong absorption band centered at 270 nm and assigned to BTZ was observed for BTZ/Fe^2+^@BTF‐DA, and the BTZ loading capacity of the nanoparticles was calculated to be 8.2% (Figure S7, Supporting Information). BTZ/Fe^2+^@BTF‐DA was also shown to emit an intense NIR‐II fluorescence at 1100–1300 nm upon excitation at 1064 nm (Figure 1J).

**Figure 2:**
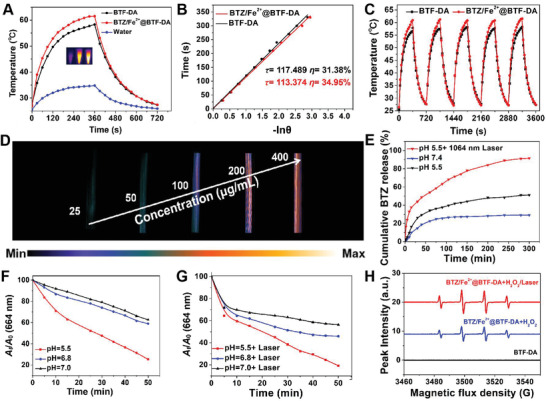
A) Photothermal heating curves of water, BTF‐DA, and BTZ/Fe^2+^@BTF‐DA (0.1 mg mL^−1^) under 1064 nm (1 W cm^−2^) laser irradiation, respectively. Inset: corresponding infrared thermographs after 6 min of irradiation. B) Cooling time versus the negative natural logarithm of the driving force temperature. C) Photothermal stability of BTF‐DA and BTZ/Fe^2+^@BTF‐DA over five on–off cycles. D) In vitro PA images of BTZ/Fe^2+^@BTF‐DA at different concentrations acquired under 1064 nm laser irradiation (1 W cm^−2^). E) Cumulative release of BTZ from BTZ/Fe^2+^@BTF‐DA over time under different conditions (pH 7.4, pH 5.5, and pH 5.5 with 1064 nm laser irradiation), Error bars, mean ± SD (*n* = 5). Hydroxyl radical (·OH) generation by BTZ/Fe^2+^@BTF‐DA under different pH conditions (pH 7.4, pH 6.8, and pH 5.5) F) with or G) without 1064 nm laser irradiation (1 W cm^−2^); ·OH generation was quantified from the decrease in the absorbance of MB at 664 nm ([BTZ/Fe^2+^@BTF‐DA] = 100 µg mL^−1^, [H_2_O_2_] = 0.5 mm, [MB] = 1 mm), Error bars, mean ± SD (*n* = 5). H) ESR spectra of aqueous solutions of BTF‐DA and BTZ/Fe^2+^@BTF‐DA under different conditions.

The obtained BTZ/Fe^2+^@BTF‐DA strongly absorbed at 1064 nm and exhibited excellent NIR‐II PTT performance. The PA amplitudes of the nanoparticles under 1064 nm laser irradiation were therefore further determined. As shown in **Figure** **2**A, upon irradiation at 1064 nm (1 W cm^−2^, 6 min), BTZ/Fe^2+^@BTF‐DA exhibited an excellent photothermal capability, inducing an increase in the aqueous temperature to 61.5 °C, whereas, under the same conditions, BTF‐DA raised the temperature to 58.3 °C. Furthermore, the photothermal conversion efficiency *η* of BTZ/Fe^2+^@BTF‐DA (34.95%) was found to be slightly higher than that of BTF‐DA (31.38%), probably because of the increased intermolecular interaction after iron chelation (Figure 2B). Furthermore, the photothermal effect of BTZ/Fe^2+^@BTF‐DA was concentration‐dependent (Figures S8 and S9, Supporting Information). BTZ/Fe^2+^@BTF‐DA is reasonably thermostable: the maximum solution temperature remained at 61.5 °C after five heating–cooling cycles (Figure 2C). BTZ/Fe^2+^@BTF‐DA emitted bright PA signals, and the intensities of the NIR‐II PAI signals increased linearly with concentration (Figure 2D and Figure S10, Supporting Information). These NIR‐II FI and NIR‐II PAI characteristics ensure that BTZ/Fe^2+^@BTF‐DA possesses an excellent potential for tumor detection via optical imaging. Subsequently, the release profiles of BTZ at different pH levels, and with or without 1064 nm laser irradiation, were investigated. As shown in Figure 2E, only 29% of the BTZ was released within 6 h at pH 7.4, demonstrating the stability of BTZ/Fe^2+^@BTF‐DA at this pH. However, under acidic conditions (pH 5.5),^[^ ^41^ ^]^ an accelerated release, with over 50% cumulative release of BTZ, was observed. Thus, the pH‐responsive decomposition of BTZ was demonstrated. Moreover, after 1064 nm laser irradiation, the release rate of BTZ is substantially elevated for BTZ/Fe^2+^@BTF‐DA at pH 5.5, suggesting that this substance also exhibits photothermal responsiveness. Hence, due to the protection of the drug molecule by the encapsulating structure, and because of the pH/photothermal‐responsive release properties of BTZ, BTZ/Fe^2+^@BTF‐DA might be expected to remain stable during blood circulation and then decompose at tumor sites under 1064 nm laser irradiation. In order to study the ·hydroxyl radical (·OH) species generation resulting from the Fenton catalytic activity of BTZ/Fe^2+^@BTF‐DA in H_2_O_2_, the catalytic efficiency was quantified using the ·OH indicator methylene blue (MB) by measuring the absorption decrease at 664 nm. In the presence of H_2_O_2_, along with a decrease in the pH from 7 to 5.5, a gradual decrease in MB absorption was noted (Figure 2F). The MB bleaching results confirmed that ·OH generation by BTZ/Fe^2+^@BTF‐DA was pH‐dependent. Furthermore, efficient production of ·OH was demonstrated to be positively correlated with 1064 nm laser irradiation. After 10 min of laser irradiation, MB decomposition by BTZ/Fe^2+^@BTF‐DA increased from 8.2 to 30.1% at pH 7.4, 13.3 to 35.6% at pH 6.8, and 29.1 to 40.4% at pH 5.5 (Figure 2G). Finally, the generation of ·OH by BTZ/Fe^2+^@BTF‐DA was detected by electron spin resonance (ESR) spectroscopy using 5,5‐dimethyl‐1‐pyrroline *N*‐oxide (DMPO) as a trap (Figure 2H). The ·OH signals were observed in the presence of BTZ/Fe^2+^@BTF‐DA and H_2_O_2_ at pH 5.5. In particular, under 1064 nm laser irradiation, stronger ·OH signals were produced in the ESR spectrum, demonstrating that hyperthermia accelerated the Fenton‐like catalytic reaction. In tumor tissue, the pH is much lower than in normal tissue; therefore, in response to the relatively low pH in tumor microenvironments and the photothermal effect, the BTZ release rate and ·OH production rate can be dramatically accelerated, which should help to avoid undesired side effects resulting from drug release away from the irradiated tumor site.

### 2.2 In Vitro Anticancer Effects of BTZ/Fe^2+^@BTF‐DA

**Figure 3:**
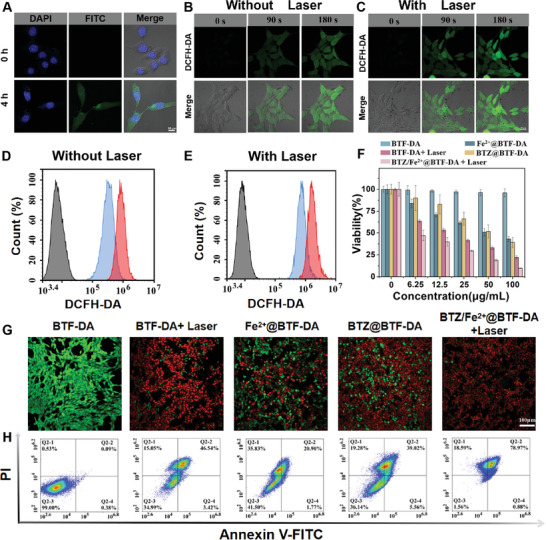
A) Cell uptake and in vitro antitumor capacity of BTZ/Fe^2+^@BTF‐DA. CLSM imaging of 4T1 cells after co‐staining with FITC/BTZ/Fe^2+^@BTF‐DA (green) and DAPI (blue). (scale bar: 10 µm). Intracellular ·OH levels detected by B,C) CLSM and D,E) flow cytometry using the DCF‐DA fluorescent probe with or without 1064 nm laser irradiation (1 W cm^−2^) (scale bars: 20 µm). F) 4T1 cell viability after incubation with BTF‐DA, BTZ@BTF‐DA, Fe^2+^@BTF‐DA, and BTZ/Fe^2+^@BTF‐DA at different concentrations with or without 1064 nm laser irradiation (1 W cm^−2^). Error bars, mean ± SD (*n* = 5). G) Live/dead assays and H) flow cytometry detection of 4T1 cells with different treatments (scale bars: 100 µm).

We first examined the intracellular behavior of BTZ/Fe^2+^@BTF‐DA in 4T1 cells by in vivo NIR‐II FI. After 4 h of incubation, the NIR‐II FI signal intensities were obviously increased (Figure S11, Supporting Information). In addition, the BTZ/Fe^2+^@BTF‐DA nanoparticles were also loaded with fluorescein isothiocyanate (FITC), and the cell uptake was detected by observing the FITC fluorescence signal. As shown in **Figure** **3**A, bright green fluorescence was observed at 4 h, indicating efficient cellular internalization of BTZ/Fe^2+^@BTF‐DA. The catalytic activity of BTZ/Fe^2+^@BTF‐DA was visualized in 4T1 cells. To specifically evaluate the intracellular ·OH level, a fluorescent turn‐on probe, 2′,7′‐dichlorofluorescin diacetate (DCF‐DA), was employed as the ·OH indicator. Images of 4T1 cells incubated with BTZ/Fe^2+^@BTF‐DA were acquired using confocal laser scanning microscopy (CLSM). Bright green fluorescence was observed in the cells after treatment with BTZ/Fe^2+^@BTF‐DA (Figure 3B). This result confirmed the specific microenvironment‐responsive catalytic activity of BTZ/Fe^2+^@BTF‐DA. More importantly, upon 1064 nm laser irradiation, the intensity of the green fluorescence of the cancer cells incubated with BTZ/Fe^2+^@BTF‐DA was significantly greater than that observed before laser irradiation (Figure 3C), demonstrating that the photothermal effect results in substantially elevated ·OH levels in tumor cells. To further investigate the H_2_O_2_ generation and photothermal responsivity of the intracellular ·OH release, flow cytometry analysis was used to examine the intensity of the green fluorescence signal. The flow cytometry analysis results revealed a significant rise in the green fluorescence intensity in the 4T1 cancer cells incubated with BTZ/Fe^2+^@BTF‐DA with and without laser irradiation, respectively, compared with those incubated with BTF‐DA (Figure 3D,E), which is consistent with the CLSM imaging observations.

The in vitro antitumor capability of BTZ/Fe^2+^@BTF‐DA was verified for 4T1 cells via a cell counting kit‐8 (CCK‐8) analysis. Under 1064 nm laser irradiation, BTZ/Fe^2+^@BTF‐DA played a synergistic NIR‐II PTT/chemotherapy/CDT role, resulting in the lowest 4T1 cancer cell viability among the various nanoparticle treatments (Figure 3F). However, in the individual NIR‐II PTT (BTF‐DA+1064 nm laser), chemotherapy (BTZ@BTF‐DA), and CDT (Fe^2+^@BTF‐DA+1064 nm laser) groups, only moderate cell death was induced. As expected, neither the untreated cancer cells nor the BTF‐DA treatment alone led to identifiable decreases in cell viability. To visually assess the therapeutic effect of BTZ/Fe^2+^@BTF‐DA, we further explored the cancer cell survival using CLSM with calcein‐AM/PI double staining.^[^ ^42^ ^]^ As depicted in Figure 3G, the 4T1 cells exhibited green fluorescence in the untreated and BTF‐DA treatment groups. In the BTZ/Fe^2+^@BTF‐DA+1064 nm laser group, most of the cells died and hence this group exhibited the brightest red fluorescence. However, in the individual treatment groups, there were obviously fewer dead cells than in the combined NIR‐II PTT/chemotherapy/CDT group. Finally, we explored the capacity of BTZ/Fe^2+^@BTF‐DA to induce apoptosis using the calcein‐AM/PI double staining method and flow cytometry. As shown in Figure 3H, at 98.44%, the cell apoptosis rate was highest for the BTZ/Fe^2+^@BTF‐DA+1064 nm laser group, and this value far exceeded those for the individual NIR‐II PTT (65.01%), chemotherapy (63.86%), or CDT (58.5%) groups. Collectively, these results demonstrate the improved anticancer effects of the synergistic BTZ/Fe^2+^@BTF‐DA treatment at the cellular level.

### 2.3 In Vivo NIR‐II FI and Anticancer Efficacy in 4T1 Mouse Xenograft Model

**Figure 4:**
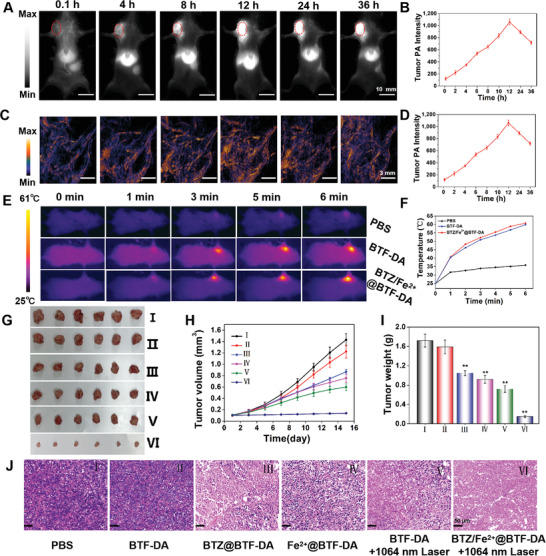
A) NIR‐II fluorescence images (scale bars: 10 mm, 1300 LP filters with 800 ms exposure time) and C) NIR‐II PA images (scale bars: 3 mm) of mice with 4T1 tumors at various time intervals after tail vein injection with BTZ/Fe^2+^@BTF‐DA (2 mg mL^−1^, 120 µL). The overlaid dashed circle indicates the tumor region. B) FI and D) PAI signal quantification in the tumor regions at different time points. E) Corresponding infrared photothermal images and F) temperature variation at the tumor sites after tail intravenous injection with PBS, BTF‐DA, and BTZ/Fe^2+^@BTF‐DA under 1064 nm laser irradiation (1 W cm^−2^, 6 min). G) Tumor images, H) tumor volume, and I) tumor weight after various treatments. Error bars, mean ± SD (*n* = 6). **p < 0.01. (I: PBS, II: BTF‐DA, III: BTZ@BTF‐DA, IV: Fe^2+^@BTF‐DA, V: BTF‐DA+1064 nm laser, VI: BTZ/Fe^2+^@BTF‐DA+1064 nm laser, 1 W cm^−2^). J) Images of H&E stained tumor tissues (scale bars: 50 µm).

Encouraged by the remarkable in vitro therapeutic performance of BTZ/Fe^2+^@BTF‐DA, the in vivo cancer inhibition efficiency was first assessed in 4T1 tumor‐bearing mice. In order to identify the optimal therapeutic time point for in vivo NIR‐II PTT/chemotherapy/CDT, the accumulation and bio‐distribution of BTZ/Fe^2+^@BTF‐DA were investigated in a 4T1 tumor‐bearing model by both NIR‐II FI and NIR‐II PAI. After intravenous injection of BTZ/Fe^2+^@BTF‐DA, the NIR‐II fluorescence signal intensity gradually increased in the tumor region, peaking at 12 h post‐injection (**Figure** **4**A,B). At this time point, the NIR‐II fluorescence signal intensity was 8.94‐fold compared with that at the initial injection time, indicating the accumulation of BTZ/Fe^2+^@BTF‐DA in the tumor tissue. Similar to NIR‐II FI results, the NIR‐II PA signal intensity progressively increased, and for the tumor site, it was almost 8.8‐fold greater at 12 h post‐injection compared to that before injection (Figure 4C,D). After 12 h, the NIR fluorescence and NIR‐II PA signals further decreased with time. Meanwhile, as shown in Figure S13, Supporting Information, the blood circulation time curves of BTZ/Fe^2+^@BTF‐DA were calculated by collecting the blood samples from SD mice at different times. Moreover, NIR‐II FI of major organs (including tumor, live, spleen, heart, and kidney) was performed 24 h post‐injection. The ex vivo NIR‐II FI results revealed that BTZ/Fe^2+^@BTF‐DA was mainly accumulated in the tumor and liver (Figure S14, Supporting Information).

The in vivo anticancer efficacy of BTZ/Fe^2+^@BTF‐DA in 4T1 tumor‐bearing mice was investigated. The tumor size was monitored every 2 days after the tumor‐bearing mice underwent intravenous administration of PBS, BTF‐DA, BTZ@BTF‐DA, Fe^2+^@BTF‐DA, or BTZ/Fe^2+^@BTF‐DA. As shown in Figure 4E,F, under 1064 nm laser irradiation (1 W cm^−2^) at 12 h post‐administration, the temperature at the tumor site for the BTZ/Fe^2+^@BTF‐DA and BTZ@BTF‐DA treatment groups increased to 60.2 and 59 °C, respectively, whereas the temperature for the PBS group increased only to 35.4 °C. The tumor growth rate of the BTF‐DA group was similar to that of the PBS group, indicating that the BTF‐DA treatment did not inhibit tumor development (Figure 4H). Compared with the PBS treatment group, the BTZ@BTF‐DA and Fe^2+^@BTF‐DA groups exhibited slight tumor growth inhibition, which indicates that neither chemotherapy nor CDT alone was able to sufficiently inhibit tumor development. Meanwhile, mice in the group treated with BTF‐DA and 1064 nm laser irradiation (NIR‐II PTT group) exhibited moderately inhibited tumor growth. By contrast, the mice subjected to the synergistic NIR‐II PTT/chemotherapy/CDT treatment (BTZ/Fe^2+^@BTF‐DA+1064 nm laser group) exhibited almost complete tumor inhibition, and the tumor was too small to extract at 14 days post‐administration (Figure 4G). At the end of the treatment period, the weights of the tumors in these groups were recorded and hematoxylin and eosin (H&E) staining was performed to examine tumor apoptosis. The tumor weight analysis indicated that the BTZ/Fe^2+^@BTF‐DA+1064 nm laser group had the highest tumor inhibition rate among all the groups (Figure 4I), which further verifies that BTZ/Fe^2+^@BTF‐DA has significant in vivo anticancer efficacy owing to a synergistic effect of NIR‐II PTT, chemotherapy, and CDT. In addition, it can be seen that in the BTZ/Fe^2+^@BTF‐DA+1064 nm laser group, the tumor tissue contained the largest regions of necrosis and the most significant apoptosis (Figure 4J).

### 2.4 In Vivo Anticancer Effect of BTZ/Fe^2+^@BTF/ALD in 4T1 Bone Metastasis Model

**Figure 5:**
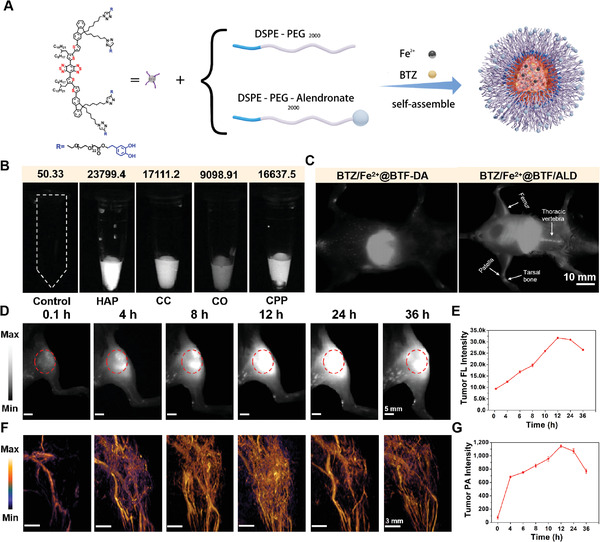
A) Schematic showing self‐assembly of BTZ/Fe^2+^@BTF/ALD. B) Calcium‐binding fluorescence image of BTZ/Fe^2+^@BTF/ALD (abbreviations: HAP, hydroxyapatite; CC, calcium carbonate; CO, calcium oxalate; CPP, calcium pyrophosphate, 1300 LP filters with 50 ms exposure time). C) In vivo bone‐specific images obtained 12 h after injection of BTZ/Fe^2+^@BTF‐DA and BTZ/Fe^2+^@BTF/ALD. Bones, including femur, patella, tarsal bone, and thoracic vertebrae, appear as bright regions in the images (scale bars: 10 mm, 1300 LP filters with 800 ms exposure time). D) FL images (scale bars: 5 mm, 1300 LP filters with 800 ms exposure time) and F) NIR‐II PA images (scale bars: 3 mm) of mice with bone metastasis models at various time intervals after tail vein injection with BTZ/Fe^2+^@BTF/ALD (2 mg mL^−1^, 120 µL). The overlaid dashed circle indicates the tumor region. Tumor E) FL and G) PA signal quantification at different time points.

ALD ligands are known to exhibit a high chelating ability for calcium ions in the bone microenvironment. Because of its bone affinity (osteotropicity), ALD has been adopted as a bone‐targeting ligand in a variety of bone therapeutic nanoagents in order to achieve drug accumulation in the bone. As shown in **Figure** **5**A, ALD‐modified DSPE‐mPEG2000 (DSPE‐mPEG2000‐ALD) and DSPE‐mPEG2000 were used to load BTF‐DA, BTZ, and Fe^2+^ ions to prepare the bone‐targeting nanoparticles BTZ/Fe^2+^@BTF/ALD. The *D*_h_ results of BTZ/Fe^2+^@BTF/ALD in aqueous solution, PBS, FBS, and DMEM were 232, 232, 232, and 231 nm, respectively, as shown in Figure S17, Supporting Information. This indicates that the nanoparticles are stable under different solution environments such as water, FBS, and DMEM. The in vitro and in vivo bone‐targeting ability of BTZ/Fe^2+^@BTF/ALD was investigated by in vivo NIR‐II FI. Hydroxyapatite (HAP) is the most abundant mineral in bone, and hence, the binding affinity of BTZ/Fe^2+^@BTF/ALD toward HAP was first evaluated as an approximate model of the in vitro bone microenvironment. Meanwhile, four other calcium salts including calcium carbonate (CC), calcium oxalate (CO), and calcium pyrophosphate (CPP) were selected as negative control groups. As illustrated in Figure 5B, the BTZ/Fe^2+^@BTF/ALD treated CC, CO, and CPP salts showed much lower NIR‐II fluorescence signals than HAP. This result indicates the superior affinity of BTZ/Fe^2+^@BTF/ALD for HAP to other inorganic compounds. BTZ/Fe^2+^@BTF‐DA and BTZ/Fe^2+^@BTF/ALD were administered intravenously to BALB/c mice, and whole‐body NIR‐II FI was performed at different time intervals after injection (Figure S19, Supporting Information). As shown in Figure 5C, areas of intense signal in the images corresponding to bones, including the femur, patella, tarsal bone, and thoracic vertebrae, were apparent for the BTZ/Fe^2+^@BTF/ALD group. At 12 h post‐injection, ex vivo NIR‐II FI of the isolated tissues from the femur, patella, tarsal bone, and thoracic vertebrae was performed. The ex vivo results (Figure S20, Supporting Information) were in accordance with the in vivo bone imaging findings, validating the excellent bone target ability of BTZ/Fe^2+^@BTF/ALD owing to the affinity of ALD for calcium ions.

**Figure 6:**
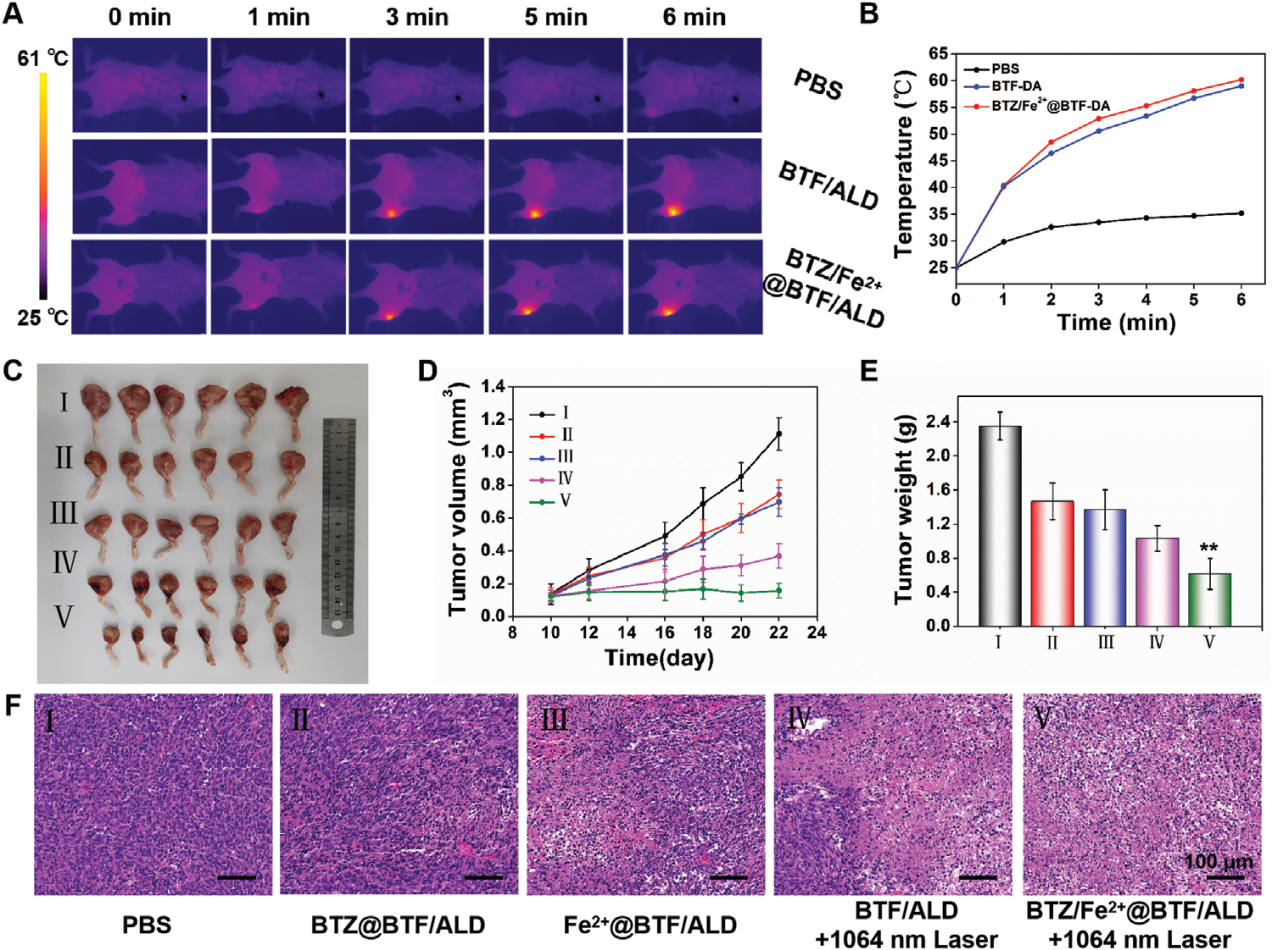
A) Corresponding infrared photothermal images and B) temperature changes at tumor sites after tail intravenous injection with PBS, BTF/ALD, and BTZ/Fe^2+^@BTF/ALD under 1064 nm laser excitation (1 W cm^−2^, 6 min). C) Photograph, D) volumes, and E) weights of tumors extracted from mice after various treatments. Error bars, mean ± SD (*n* = 6). **p < 0.01. F) Images of H&E‐stained tumor tissues (scale bars: 100 µm) (I: PBS, II: BTZ@ BTF/ALD, III: Fe^2+^@BTF/ALD, IV: BTF/ALD+1064 nm laser, V: BTZ/Fe^2+^@BTF/ALD +1064 nm laser, 1 W cm^−2^).

In view of the excellent theranostic effect of BTZ/Fe^2+^@BTF‐DA demonstrated in the 4T1 mouse xenograft model, the anti‐metastatic‐tumor activity of BTZ/Fe^2+^@BTF/ALD was further evaluated in mice using the 4T1 bone metastasis model. To investigate the tumor accumulation of BTZ/Fe^2+^@BTF/ALD, we first performed NIR‐II FI and NIR‐II PAI of the bone metastases in the mice after administration of BTZ/Fe^2+^@BTF/ALD by tail vein injection. As shown in Figure 5D,F, the intensity of the NIR‐II fluorescence and NIR‐II PA signal in the bone metastatic tumor was significantly enhanced, peaking at 12 h after injection, which demonstrates the significant bone metastatic tumor‐targeting ability of BTZ/Fe^2+^@BTF/ALD. Impressively, ex vivo NIR‐II FI of harvested tibia bone metastatic tumors and other organs demonstrate that BTZ/Fe^2+^@BTF/ALD mainly accumulated in the metastatic tumors and liver (Figure S21, Supporting Information). In addition, the bone metastasis mouse model was subsequently employed to quantitatively evaluate the synergistic NIR‐II PTT/chemotherapy/CDT treatment efficacy under 1064 nm laser irradiation using BTZ/Fe^2+^@BTF/ALD. As displayed in **Figure** **6**A,B, the maximum temperature of the bone metastatic tumors increased to 60.9 °C, as a result of laser irradiation at 1064 nm at 12 h after intravenous injection of BTZ/Fe^2+^@BTF/ALD; this is a significant temperature increase compared to that observed for the PBS group. Not surprisingly, the tumor growth rate was significantly suppressed in the BTZ/Fe^2+^@BTF/ALD+1064 nm laser group (Figure 6C,D), and the suppression rate was larger than that in the NIR‐II PTT treatment group (BTF/ALD+1064 nm laser), chemotherapy treatment group (BTZ@BTF/ALD), and CDT treatment group (Fe^2+^@BTF/ALD). At the end of the treatment period, significantly reduced metastatic tumor weights were observed in the BTZ/Fe^2+^@BTF/ALD+1064 nm laser group compared to those in the control groups (Figure 6E). This remarkable anti‐metastatic tumor efficacy for BTZ/Fe^2+^@BTF/ALD was attributed to its bone‐targeting ability and the combination of NIR‐II PTT, chemotherapy, and CDT. Furthermore, H&E staining of the metastatic tumor showed that more histopathological damage occurred in the BTZ/Fe^2+^@BTF/ALD+1064 nm laser group than in the three other groups (Figure 6F). The above tumor volume, tumor weight, and cellular dissociation data results verify that BTZ/Fe^2+^@BTF/ALD has excellent antitumor efficacy for bone metastatic tumors. As such, this phototheranostic platform BTZ/Fe^2+^@BTF/ALD shows the following unique advantages: 1) This platform uses the boronate–catechol linkage and coordination interactions to enhance the BTZ drugs and Fe^2+^ ions loading capacity; 2) Under acidic conditions and hydrogen peroxide (H_2_O_2_) over expression tumor microenvironment,^[^ ^43^, ^44^ ^]^ the BTZ and Fe^2+^ ions are released to initiate the chemotherapy and CDT; 3) The hyperthermia generated by NIR‐II irradiation of not only implements NIR‐II PTT but also accelerates BTZ release and the Fenton reaction. Therefore, the combination of NIR‐II PTT, BTZ chemotherapy, and CDT raises multiple anticancer methods and this phototheranostic platform has a synergistic effect; 4) ALD‐coated platform can achieve fine targeting toward bone, promoted the BTZ/Fe^2+^@BTF/ALD to reach the site of 4T1 cells bone metastasis and the accumulation in breast cancer.

Furthermore, negligible changes in body weight were detected in all the tested groups during the entire therapeutic process (Figure S22, Supporting Information), and negligible damage was found in the major organs (including the heart, spleen, liver, and kidney) after synergistic NIR‐II PTT/chemotherapy/CDT treatment (Figure S23, Supporting Information), suggesting that BTZ/Fe^2+^@BTF/ALD might have very few side effects. Finally, in order to evaluate the potential toxicity of BTZ/Fe^2+^@BTF/ALD, hematology and biochemistry analyses were conducted using healthy mice after intravenous injection of BTZ/Fe^2+^@BTF/ALD. The parameters given in Figure S24, Supporting Information, indicate that the mice injected with BTZ/Fe^2+^@BTF/ALD did not exhibit any significantly abnormal phenomena, demonstrating that BTZ/Fe^2+^@BTF/ALD is biocompatible.

## 3 Conclusion

In summary, a NIR‐II (1000–1700 nm) excitation BTZ/Fe^2+^@BTF/ALD phototheranostic nanoagent was successfully developed for synergistic NIR‐II PTT/chemotherapy/CDT treatment of breast cancer bone metastases. BTZ/Fe^2+^@BTF/ALD was shown to possess intense NIR‐II absorption, excellent NIR‐II fluorescence emission, and outstanding photothermal conversion efficiency (*η* = 34.95%) properties, as well as produce a NIR‐II PA signal, accelerate BTZ release, and increase the Fenton reaction rate, under 1064 nm light irradiation. More importantly, BTZ/Fe^2+^@BTF/ALD exhibited excellent bone targeting ability, and significant accumulation in breast cancer bone metastases was shown. This NIR‐II light‐triggered phototheranostic was shown not only to allow accurate NIR‐II FI/NIR‐II PAI dual‐modality tracking in vivo, but it also effectively inhibited tumor growth in both murine 4T1 tumor xenograft and murine 4T1 bone metastasis models. Consequently, this NIR‐II excitation phototheranostic platform represents an exciting new approach to the treatment of breast cancer bone metastases.

## Conflict of Interest

The authors declare no conflict of interest.

## Supporting information

Supporting Information

## Data Availability

The data that support the findings of this study are available from the corresponding author upon reasonable request.
