# Supplementary material for: NIR‐II Excitation Phototheranostic Platform for Synergistic Photothermal Therapy/Chemotherapy/Chemodynamic Therapy of Breast Cancer Bone Metastases
Source: Adv Sci (Weinh). 2022 Oct 10;9(33):2204718. doi: 10.1002/advs.202204718 (PMC9685450; doi:10.1002/advs.202204718)
Supplement: Supplementary file 1 — Supporting Information [file ADVS-9-2204718-s001.pdf]

ADVANCED SCIENCE
Open Access

Supporting Information

for *Adv. Sci.*, DOI 10.1002/advs.202204718

NIR-II Excitation Phototheranostic Platform for Synergistic Photothermal Therapy/Chemotherapy/Chemodynamic Therapy of Breast Cancer Bone Metastases

Pengfei Sun, Fan Qu, Chi Zhang, Pengfei Cheng, Xiangyu Li, Qingming Shen, Daifeng Li and Quli Fan**

Supporting Information

NIR-II Excitation Phototheranostic Platform for Synergistic Photothermal Therapy/Chemotherapy/Chemodynamic Therapy of Breast Cancer Bone Metastases

Pengfei Sun[1,a] , Fan Qu[1,a], Chi Zhang[2,a], Pengfei Chen[1], Xiangyu Li[1], Qingming Shen[1], Daifeng Li,[2] and Quli Fan[1*]*

Table of Contents

Experimental section 3
Figure S1. ^{1}H NMR spectrum of BBT-FT in $CDCl_3$. 15
Figure S2. ^{1}H NMR spectrum of BBT-FT-N_3 in $CDCl_3$. 15
Figure S3. ^{1}H NMR spectrum of BBT-FT-DA in DMSO. 16
Figure S4. (A) Absorption and (B) fluorescence emission spectra of BBT-FT-DA (0.1 mg/mL) in THF. 16
Figure S5. Absorption spectra of BTZ/Fe^{2+}@BTF-DA in water. 17
Figure S6. The mole extinction coefficient of BTZ/Fe^{2+}@BTF-DA at 1064 nm absorption. 17
Figure S7. (A) Absorption spectra of BTZ/Fe^{2+}@BTF-DA in water (0.5 mg mL^{-1}). (B) Standard curve of BTZ at 270 nm. 17
Figure S8. Photothermal experiment real-time imaging of water, BTF-DA and BTZ/Fe^{2+}@BTF-DA. 18
Figure S9. Photothermal heating curves of BTF-DA and BTZ/Fe^{2+}@BTF-DA at different concentrations under 1064 nm (1.0 W cm^{-2}) laser irradiation 18
Figure S10. A linear relationship between PA signal and BTZ/Fe^{2+}@BTF-DA concentration.18
Figure S11. NIR-II fluorescence intensity of 4T1 cells incubated with or without BTZ/Fe^{2+}@BTF-DA. Insert: NIR-II fluorescence images of 4T1 cells incubated with or without BTZ/Fe^{2+}@BTF-DA. 19
Figure S12. (A) NIR-II fluorescent angiography of mice under 1064nm laser. (B) The half-peak width at the blood vessel was selected. 19
Figure S13. Blood circulation time curves of BTZ/Fe^{2+}@BTF/ALD administrated mice at various time points at 24 h post-injection. The red dashed line shows the fluorescence intensity of blood from mice without any treatment (control). 19
Figure S14. NIR-II fluorescence images of tumors and viscera of mice with 4T1 tumors after dissection. 20
Figure S15. Body weight of mice with 4T1 tumor under different treatment (I: PBS, II: BTF-DA, III: BTZ@BTF-DA, IV: Fe^{2+}@BTF-DA, V: BTF-DA+1064 nm Laser, VI: BTZ/Fe^{2+}@BTF-DA+1064 nm Laser, 1.0 W cm^{-2}). 20
Figure S16. H&E staining images of major organs in 4T1 tumor mice (I: PBS, II: BTF-DA, III: BTZ@BTF-DA, IV: Fe^{2+}@BTF-DA, V: BTF-DA+1064 nm Laser, VI: BTZ/Fe^{2+}@BTF-DA+1064 nm Laser, 1.0 W cm^{-2}). 21
Figure S17. Particle size of BTZ/Fe^{2+}@BTF/ALD in aqueous solution, PBS, FBS and DMEM. 21
Figure S18. (a) Absorption and (b) fluorescence emission spectra of BTZ/Fe^{2+}@BTF/ALD (0.1 mg/mL) in aqueous solution 21

Figure S19. Bone targeted fluorescence imaging at different time intervals. 22
Figure S20. NIR-II fluorescence image of isolated organs and bones from normal mice injected with BTZ/Fe^{2+}@BTF/ALD. 22
Figure S21. NIR-II fluorescence images of tumor and visceral NIR-II after dissection in a mouse model with bone metastases. 23
Figure S22. Body weight of mice with bone metastasis model under different treatment (I: PBS, II: BTF-DA, III: BTZ@BTF-DA, IV: Fe^{2+}@BTF-DA, V: BTF-DA+1064 nm Laser, VI: BTZ/Fe^{2+}@BTF-DA+1064 nm Laser, 1.0 W cm^{-2}).. 23
Figure S23. Image of major organs stained with H&E in a mouse model of bone metastases under treatment of BTZ/Fe^{2+}@BTF-DA+1064 nm Laser. 24
Figure S24. Blood analysis of the mice (I: PBS, II: BTF-DA, III: BTZ@BTF-DA, IV: Fe^{2+}@BTF-DA, V: BTF-DA+1064 nm Laser, VI: BTZ/Fe^{2+}@BTF-DA+1064 nm Laser, 1.0 W cm^{-2}).. 24
References 24

Experimental section

Materials.

4,8-Bis(5-bromo-4-(2-octyldodecy)thiophen-2-yl)-benzo[1,2-*c*;4,5-*c*']bis[1,2,5]thiadaole) (BBT, 98%) and 2-Bromo-9,9-bis(6-bromohexyl)-9*H*-fluorene were purchased from SunaTech Inc. The other reactants like dopamine (DA), N-Hydro xysuc cinimide (NHS) and (3-Dimethylaminopropyl)-3-ethylcarbodiimide hydrochloride (EDC) are going to be purchased from Macklin. ALK-PEG1000-COOH is going to be purchased from Shanghai Ponsure Biotech, Inc. Catalysts such as Tri-o-tolylphosphine, tris-(dibenzylideneacetone)dipalladium(0), triphenylphosphine, 2-(Dimethylamino)ethyl methacrylate (99%), CuTC, etc., are purchased from commercial sources (such as *Aldrich*, *J&K* Scientific Ltd., and Sigma-Aldrich). Unless indicated otherwise, all synthetic procedures were performed in an anhydrous and oxygen-free environment, and all reagents were received from commercial sources. These regents were used without further purification, except toluene, which was dried and distilled with N_2 before use. The 2-(9,9-bis(6-bromohexyl)-9H-fluoren-2-yl)-4,4,5,5-tetramethyl -1,3,2-dioxaborolane (F) and 2-(9,9-bis(6-bromohexyl)-9H-fluoren-2-yl)thiophene (FT) were prepared according to the methods used in previous methods[1]. Dulbecco's modified eagle medium (DMEM), 4T1 cells, Annexin V-FITC/propidium iodide (PI) cell apoptosis kit, Cell Counting Kit (CCK-8), 2’,7’-dichlorodihydrofluorescein diacetate (DCFH-DA) fluorescent probe and calcein-AM/propidium iodide (PI) cell apoptosis kit were achieved from KeyGEN BioTECH. Fetal bovine serum (FBS, Gibco, U.S.) was obtained from Gene Tech Co. (Shanghai, China).

Characterization. The ^{1}H NMR spectra were recorded with a Bruker Ultra Shield Plus 400 MHz spectrometer in deuterated dimethyl sulfoxide (DMSO-*d*6) or deuterated chloroform ($CDCl_3$). The morphology of nanoparticles was determined by transmission electron

microscope (HT7700, TEM) under 100 KV acceleration voltage. Dynamic light scattering (DLS) analysis using a commercial laser light scattering spectrometer (ALV-7004; ALV, Langan, Germany) is equipped with a multi- τ digital time correlator and a He-Ne laser (at λ = 632.8 nm). The $\langle D_h \rangle$ data was extracted by CONTIN analysis. All samples we used for testing were optically cleared with a Millipore filter (0.45 μm). Tests were performed at room temperature at a scattering Angle of 90° . Scanning transmission electron microscopy-energy dispersive X-ray (STEM-EDX) element mapping was performed on JEOL 2100F with ultra-high resolution (UHR) confifiguration (accelerating voltage: 200 kV). NIR-II fluorescence spectra were measured using an NIR-II spectrophotometer (Fluorolog 3, Horiba). NIR InGaAs was selected as the detector, with an excitation wavelength of 1064 nm obtained from a diode laser operating at 25.0 ± 0.5 °C. After the raw emission data were collected, the fluorescence signal was further confirmed and corrected for the sensitivity of InGaAs detector profile and output through the T1c channel. The laser was purchased from Changchun New Industries Optoelectronics Technology Co., Ltd. The in vitro and in vivo NIR-II FI experiments were conducted on an NIR-II imaging system (Wuhan Grand-imaging Technology Co., Ltd) with 1064 nm LP and 1200 LP filters and two types of lenses (50 or 100 mm) under the 808 nm or 1064 nm laser irradiation. A 640 × 512 pixel two-dimensional InGaAs array from Princeton Instruments in NIR-II fluorescence windows was equipped in this NIR-II FI system. All photothermal tests were detected using a Fotric 225 instrument (IR thermal camera, ± 2 °C) purchased from Fotric. (Shanghai, China). The Cell Counting Kit-8 (CKK-8) assay was carried out on A PowerWave XS/XS2 microplate spectrophotometer (BioTek, Winooski, VT). All *in vitro*/*in vivo* mice photoacoustic experiments were performed using the NIR-I & NIR-II small animal whole body 3D photoacoustic imaging system, model LOIS-3D/LOIS-3D Plus, manufactured by TomoWave Laboratories, USA.

Synthesis of BBT-FT. n-BuLi (1.6 M in hexane, 2.6 mL, 4.18 mmol) was added dropwise to a solution of FT (2.0 g, 3.48 mmol) in THF (20 mL) at -78 °C under N_2 protection. After stirring at -78 °C for 1 h, tributyltin chloride (1.1 mL, 4.18 mmol) was dissolved in the solution. Then, the temperature of the reaction was slowly warmed to room temperature and stirred overnight. Finally, the reaction was quenched with H_2O and extracted twice with DCM. The mixed organic phase was dried with sodium sulfate and evaporated under a vacuum without further purification. The solution of the crude product (400 mg, 0.46 mmol) mentioned above, BBT (250 mg, 0.23 mmol), $Pd_2(dba)_3$ (5 mg) and *trio*-tolylphosphine (32 mg) in anhydrous toluene (15 mL) was stirred at 100 °C for 24 h under N_2 protection. After cooling, the resulting product was evaporated and purified using column chromatography to afford the product BBT-FT (351.84 mg, Yield: 73.46%). ^1H NMR (400 MHz, $CDCl_3$) δ: 0.86-0.87 (m, 12H), 1.15-1.43 (m, 88H), 1.675-1.712 (m, 10H), 2.04-2.08 (t, 8H), 2.96-2.98 (d, 4H), 3.29-3.32 (t, 8H), 7.37-7.45 (m, 10H), 7.63-7.75 (d, 8H), 8.94 (s, 2H).

Synthesis of BBT-FT-N3. The above products BBT-FT (300 mg, 0.15 mmol) and NaN_3 (113.37 mg, 1.74 mmol) were placed in a flask, the reactants were dissolved with DMF, heated to 50 ℃ in an oil bath and reacted for 6 hours. After cooling, the resulting product was evaporated and purified using column chromatography to afford the product BBT-FT-N_3. (164 mg, Yield: 56.37%). ^1H NMR (400 MHz, $CDCl_3$) δ: 0.86-0.87 (m, 12H), 1.15-1.45 (m, 98H), 2.04-2.06 (m, 8H), 2.97-2.98 (d, 4H), 3.14-3.17 (d, 8H), 7.37-7.45 (m, 10H), 7.63-7.75 (d, 8H), 8.94 (s, 2H).

Synthesis of BBT-TF-DA. The above products BBT-FT-N_3 (160 mg, 0.08mmol) and ALK-PEG1000-COOH (380 mg, 0.35mmol) were placed in a flask, the reactants were dissolved with THF, CuTC (5 mg) was used as the catalyst, and then stirred at room temperature for 8 hours under N_2 protection. The resulting product was dialyzed in pure water with a 3500Da dialysis bag to remove unreacted impurities, and then the product obtained by freeze-drying

was BBT-FT-PEG-COOH (345mg, 68%). The BBT-FT-PEG-COOH (200mg) obtained from the reaction was dissolved in Mes Buffer (pH=6.0) , then add NHS (16mg, 0.14mmol) and EDC (26.65mg, 0.14mmol) to active the carboxyl group. The mixture was stirred for 30min at room temperature in a light-free environment. After 30min, dopamine hydrochloride was quickly added, and the mixture was stirred for 24 hours in a light-free environment with deoxygenation. The product obtained at this time was removed with a 3500Da dialysis bag to remove the unreacted magazine, and finally freeze-dried to obtain BBT-TF-DA (182.45mg, 85.85%).

Preparation of BTF-DA. Firstly, 1mg BBT-TF-DA was dissolved in 1 mL THF solution, and 10 mg DSPE-PEG2000 was dissolved in 10 ml water. THF solution containing BTF-TF-DA (1 mg mL^{-1}) was rapidly injected into 10 mL DSPE-PEG2000 aqueous solution. Continuous ultrasonic treatment was performed for 3.5 min. After nanoprecipitation occurred, THF was removed by rotary evaporation at 40 ℃ under vacuum. The aqueous solution was filtered through a polyether sulfone (PES) syringe driven filter (0.8 μm) (micropore) to remove impurities and large particles. The filtered aqueous solution was washed three times with a 30K centrifugal filtration unit (microwell) and centrifuged at 2500 rpm for 10 min. The final BTF-DA solution was stored at 4 ℃ for further use.

Preparation of BTZ@BTF-DA. Firstly, 1mg bortezomib (BTZ) and 1mg BTF-TF-DA were dissolved in 1mL THF solution, respectively. Then BTZ solution (0.5 ml , 1 mg mL^{-1}) and BTF-TF-DA solution (1 ml , 1 mg mL^{-1}) were rapidly injected into DSPE-PEG2000 aqueous solution (1 mg ml^{-1}) at a ratio of 1:2, and the solution was sonicated for 3.5 min. After nanoprecipitation occurred, the operation is the same as BTF-DA.

Preparation of Fe^{2+}@BTF-DA. The freshly prepared ferrous sulfate solution (0.1M, 0.5 mL) was dropped into pre-prepared BTF-DA (1 ml) and stirred vigorously. After continuous

agitation overnight, excess ferrous ions were removed by ultra-centrifugation at 2500 rpm for 25 min and then rinsed with water three times. Then the purified Fe^{2+}@BTF-DA was diluted and filtered through a 220nm PVDF syringe driven filter, and the Fe^{2+}@BTF-DA stock solution was obtained by ultra-centrifugation.

Preparation of BTZ/Fe^{2+}@BTF-DA. The freshly prepared ferrous sulfate solution (0.1M, 0.5 mL) was put into the prefabricated BTZ/BTF-DA (1ml) and vigorously stirred overnight. The preparation method was referred to Fe^{2+}@BTF-DA.

The loading capacity of bortezomib in BTZ/Fe^{2+}@BTF-DA. In order to evaluate the loading capacity of bortezomib in BTZ/Fe^{2}@BTF-DA, several different concentrations of tetrahydrofuran solution of bortezomib were firstly prepared and detected by UV spectrophotometer to get the absorption intensities at 270 nm, and then make a standard curve with them as shown in Figure S10. Then, 10 mL aqueous solution of BTZ/Fe^{2+}@BTF-DA with different concentrations (0.10 mg mL^{-1}, 0.05 mg mL^{-1}, 0.025 mg mL^{-1}, 0.0125 mg mL^{-1} and 0.00625 mg mL^{-1}) were prepared respectively. The above five solutions were lyophilized and respectively dissolved in 10 mL THF. The 270 nm absorbance of bortezomib before and after loading with BTZ/Fe^{2+}@BTF-DA were measured and used to calculate the bortezomib loading capacity with the standard curve according to the Beer-Lambert law. The calculated loading capacity by the UV method is about 8.2%.

***In vitro* photothermal effect and photothermal conversion efficiency.** To evaluate the photothermal effect of BTF-DA and BTZ/Fe^{2+}@BTF-DA, 200 μL of two nanoparticle solutions with concentrations of 0.1, 0.08, 0.05, and 0.025 mg mL^{-1} were successively irradiated with 1064 nm laser (1.0 W cm^{-2}, 6 min). The temperature changes of the BTF-DA and BTZ/Fe^{2+}@BTF-DA solutions were performed with an IR thermal camera, and these data were recorded every 30 s.

To study the photothermal conversion behavior of BTF-DA and BTZ/Fe^{2+}@BTF-DA, a thermal imaging camera (Fotric 225, Fotric Precision Instruments, USA, ± 2 °C) was used to perform the thermal imaging of NPs in an aqueous solution. First, BTF-DA (0.1 mg mL^{-1}) and BTZ/Fe^{2+}@BTF-DA (0.1 mg mL^{-1}) were prepared , and 200 μL of each nanoparticle was added into a 250 μL centrifuge tube. Second, the temperature changes of the fixed concentration of BTF-DA and BTZ/Fe^{2+}@BTF-DA (0.1 mg mL^{-1}) were irradiated with a 1064 nm laser (1.0 W cm^{-2}, 6 min), and then the laser was shut off. Finally, we can obtain a temperature increase and drop curve.

The photothermal conversion efficiency (ŋ) was calculated using equations (1) and (2) expressed below. The photothermal conversion efficiency of the BTF-DA and BTZ/Fe^{2+}@BTF-DA were determined to be through the collected data and equation.

$$ŋ = [hS(T_{max}-T_{suur})-Q_{dis}]/[I(1-10^{-A1064})] \qquad (1)$$

$$\tau_s = m_D C_D/hS \qquad (2)$$

The parameters S, h, T_{max}, T_{surr}, Q_{dis}, I and A_{1064} are the container's surface area, heat-transfer coefficient, maximum laser-trigger temperature, indoor temperature, heat dissipation caused by the light absorbing of quartz cuvette, intensity of laser (1.0 W cm^{-2}) and absorbance of BTF-DA and BTZ/Fe^{2+}@BTF-DA at 1064 nm, respectively.

Parameter τ_s is the time constant of the sample system. The parameters m_D and C_D are the mass and heat capacity of the solvent, respectively.

***In vitro* BTZ release.** A certain amount of BTZ/Fe^{2+}@BTF-DA was put into a 3500 Da dialysis bag, and the dialysis bag was soaked in an aqueous solution of different pH (pH=7.4 and pH=5.5). The content of BTZ in buffer solution was tested by UV at regular intervals. In addition, to detect the effects of laser irradiation, a dialysis bag containing BTZ/Fe^{2+}@BTF-

DA was immersed in an aqueous solution of pH5.5 and irradiated with 1064 nm laser (1.0 W cm^{-2}).

***In vitro* ·OH detection.** Firstly, aqueous solutions with different pH values (pH = 7.4, 6.8 and 5.5) were prepared, each solution (150 μL) was added to the 96-well plate, and H_2O_2 (50 μL, 0.5 mM) and MB (50 μL, 1mM) were added to each well, and the mixed solution was used as the test environment. Then, BTZ/Fe^{2+} @BTF-DA (0.1mg ml^{-1} 100 μL) was added to each well. Finally, the absorbance of each well was measured by PowerWaveXS/XS2 microplate spectrophotometer at 664nm at room temperature, and ·OH formation was quantified by the decrease of MB absorbance at 664nm.

Cell Culture. 4T1 breast cancer cells were cultured with Dulbecco's Modified Eagle's Medium (DMEM) supplemented with 10% fetal bovine serum (FBS) in a humidified 5% CO_2 environment at 37 °C.

***In vitro* Cellular Uptake.** The 4T1 cells were used for the assessment of cellular uptake. First, cells, seeded 4×10^4 per well, were incubated in DMEM in a 6-well plate at 37 °C and 5% CO_2 condition for 24 h. Subsequently, the medium was replaced by one of the two groups, including 1 mL fresh DMEM medium alone and the 1mL mixture of fresh DMEM medium and BTZ/Fe^{2+}@BTF-DA (0.1 mg mL^{-1}), respectively. The treated cells were further incubated at 37 °C and 5% CO_2 condition for 4 h. Afterward, the upper supernatant was sucked out and 1 mL of PBS was added to clean twice and remove dead cells. Subsequently, PBS was removed and 500 μL trypsin digestion solution with EDTA was added, and the cells were dissolved in an incubator at 37 °C and 5% CO_2 for 90 s. Later 1 mL of DMEM was added and the cells were transferred into a 15 mL centrifuge tube and centrifuged for 3 min. The supernatant was then removed, and 100 μL of PBS was added to the centrifuge tube. Finally,

the cells were transferred to a 96-well plate, where NIR-II fluorescence images of the two groups were captured under the 1064 nm laser excitation.

Cellular internalization. To track internalization of BTZ/Fe^{2+}@BTF-DA in tumor cells, 4T1 cells (1×10^4) were grown on slices in a 6-well culture plate at 37 °C and 5% CO_2 condition for 24 h, and then incubated with FITC/BTZ/Fe^{2+}@BTF-DA (0.1 mg mL^{-1}) . FITC was used as a fluorescent marker to determine whether the material was uptaken by cells. After 4 h, the cells were washed with PBS for three times, followed by costaining with DAPI (nucleus indicator, 10 min). Then, the cells were imaged by CLSM (Olympus Fluoview FV1000).

***In Vitro* Cytotoxicity Assay.** 4T1 cells were cultured with DMEM supplemented with 10% FBS. The surrounding environment is at 37 ℃ with a humidified 5% CO_2. Cells (1×10^4 cells/well) were incubated in a 96-well plate for 24 h, different concentrations of BTF-DA (100 μL) was diluted in DMEM and added to the wells, respectively. Cells were cultured for another 24 h. After that, each well of the microliter plate was added with 10 μL of Cell Counting Kit-8 (CCK-8) solution and the plate was cultured for accessional 30 min in CO_2 incubator. Finally, use the Bio-tek Synergy HTX microplate spectrophotometer to determine the 450 nm absorbance of each well. The following formula was used to calculate the viability of cell growth: Viability (%) = (mean absorbance value of treatment group/mean absorbance value of control group) × 100%.

Intracellular ROS detection by confocal imaging and flow cytometry. The intracellular ROS molecule was detected using a ROS probe (DCFH-DA). Two group 4T1 cells were inoculated in a cell culture dish and incubated with complete DMEM (containing 10% FBS) for 24 h under standard condition (37℃, 5% CO_2) in dark. Then, BTZ/Fe^{2+}@BTF-DA was added to fresh DMEM to obtain the mixed medium (0.1 mg mL^{-1}). After the 4T1 cells had been co-incubated with this mixed medium for 4 h in the dark, the media were removed and

cells were washed twice with 1 mL PBS solution, then 1 mL DCFH-DA was added to stain cells for 25 min. After fixation, cells were washed two times with 1 mL PBS solution and 1 mL fresh DMEM was added to the cell culture dish. Then, one group was irradiated with a 1064 nm laser with a power of 1 W cm^{-2}, and the other group was irradiated without laser. After 0, 90 and 180 s, the cells were imaged with CLSM (Olympus Fluoview FV1000), respectively. In addition, ROS detection was confirmed by flow cytometry.

Assessment of NIR-II Photothermal/Chemotherapy/Chemodynamic therapy Synergistic Therapies *in vitro* Using the CKK-8 assay. For the cell viability analysis, 4T1 cells were first cultured in 96-well plates at a density of 2×10^4 cells per well. After 24 h, cells were transferred to a mixed medium containing different doses (0, 6.25, 12.5, 25, 50, and 100 mg μL^{-1}) of materials from different groups (BTZ@BTF-DA, Fe^{2+}@BTF-DA, BTF-DA + Laser, BTZ/Fe^{2+}@BTF-DA + Laser) and incubated in the dark. Thereafter, selected wells from the different groups were illuminated with or without an 1064 nm laser at a power of 1 W cm^{-2} for 6 min. After another incubation for 24 h, 100 μL fresh DMEM and 10 μL of Cell Counting Kit-8 (CCK-8) solution were added to each well and incubated for another 30 min. Thereafter, the 96-well plates were shaken for 10 min. Finally, the absorbance of each well was measured at 450 nm using a PowerWave XS/XS2 microplate spectrophotometer.

Assessment of NIR-II Photothermal/Chemotherapy/Chemodynamic therapy Synergistic Therapies effect *in vitro* by confocal imaging. 4T1 cells were cultured with DMEM in CLSM culture dishes (Costar) until the cell density increased to 1×10^5 cells mL^{-1} per well. Materials in different groups (BTF-DA, BTZ@BTF-DA, Fe^{2+}@BTF-DA, BTF-DA + Laser, BTZ/Fe^{2+}@BTF-DA + Laser) were added to fresh DMEM to obtain the mixed medium (0.1 mg mL^{-1}). After the 4T1 cells had been co-incubated with this mixed medium for 4 h in the dark, the media were removed, the cells were washed by PBS and replaced with fresh DMEM. Next, the selected wells from different groups were illuminated with or without a 1064 nm

laser at a power of 1 W cm^{-2} for 6 min. After a 12 h incubation to allow cells to undergo apoptosis, the medium were removed and the cells were incubated with Calcein AM/propidium iodide (PI) solution for 30 min. Next, the cells were washed by PBS and replaced with 1 mL fresh DMEM. Then, the cells were imaged by CLSM (Olympus Fluoview FV1000).

Assessment of NIR-II Photothermal/Chemotherapy/Chemodynamic therapy Synergistic Therapies *in vitro* Using Flow Cytometry. 4T1 cells were cultured with DMEM in 6-well plates until the cell density increased to 1×10^5 cells mL^{-1} per well. Materials in different groups (BTF-DA, BTZ@BTF-DA, Fe^{2+}@BTF-DA, BTF-DA + Laser, BTZ/Fe^{2+}@BTF-DA + Laser) were added to fresh DMEM to obtain the mixed medium (0.1 mg mL^{-1}). After the 4T1 cells had been co-incubated with this mixed medium for 4 h in the dark, the media were removed and replaced with 1 mL fresh DMEM. Next, the selected wells from different groups were illuminated with or without a 1064 nm laser at a power of 1 W cm^{-2} for 6 min. After a 12 h incubation to allow cells to undergo apoptosis, the 4T1 cells that had detached from the 6-well plates were washed several times with 1 mL PBS. Subsequently, PBS was removed and 500 μL trypsin digestion solution without EDTA was added, and the cells were dissolved in an incubator at 37 °C and 5% CO_2 for 90 s. Later 1 mL of DMEM was added and the cells were transferred into a 15 mL centrifuge tube and centrifuged for 3 min. An Annexin V-FITC/PI staining solution was then mixed with the collected cells for 15 min, and cells were analyzed using flow cytometry.

Animal Experiments with 4T1 tumor. All small animal experiments are carried out in accordance with the specifications of The National Regulation of China for Care and Use of Laboratory Animals, which have been approved by the Institutional Committee on the Ethics of Animal Experiments of Zhengzhou University, Zhengzhou, China. Five or six-week-old

Balb/c mice with subcutaneous heterogeneous 4T1 tumor were achieved from the Shanghai Laboratory Animal Center with pathogen-free feeding environment.

Animal Experiments with bone metastasis models. For the bone metastasis models, tumor cells (1×107 each) suspended in a 100 μL of PBS were injected subperiosteally onto the right proximal lateral tibia. Tumor volume (mm^3) = (length + width) (mm) × length (mm) × width (mm) × 0.2618. Tumor volume was measured every other day for the duration of the treatment schedule. All animal protocols were approved by the Institutional Committee on the Ethics of Animal Experiments of Zhengzhou University, Zhengzhou, China.

***In vivo* NIR-II fluorescence imaging.** When the tumors reached a volume of 90-120 mm^3, 4T1 tumor bearing mice or bone metastasis model mice were intravenously injected with BTZ/Fe^{2+}@BTF-DA (120 μL, 1.5 mg mL^{-1}) or BTZ/Fe^{2+}@BTF/ ALD (120 μL, 1.5 mg mL^{-1}). The tumor bearing mice were imaged alive by anesthetizing them with isoflurane during the test time (just about 10 minutes) in the case of the effect of respiration. The real-time *in vivo* NIR-II fluorescence imaging was performed at diff erent post-injection times by using an *in vivo* NIR-II fluorescence imaging system. The analysis of the signal intensity of NIR-II image was performed using the NIR-II *in vivo* imaging system software.

***In vivo* NIR-II photoacoustic imaging.** When the tumors reached a volume of 90-120 mm^3, 4T1 tumor bearing mice or bone metastasis model mice were intravenously injected with BTZ/Fe^{2+}@BTF-DA (120 μL, 1.5 mg mL^{-1}) or BTZ/Fe^{2+}@BTF/ALD (120 μL, 1.5 mg mL^{-1}). Mice are mounted on specially designed holders, which rotate 360 degrees with sophisticated rotating motors. Four short pulsed laser beams (biorthogonal, bioblique) are emitted by a laser the tissue absorbs light energy to generate ultrasonic signal, which is received by a professionally designed 125° are transducer. The signal was collected at 360°, and the photoacoustic image was reconstructed by special 3D reconstruction software through unit transformation of DAQ data.

Bone targeting assay. Two mice were injected into the tail vein with BTZ/Fe^{2+}@BTF-DA (as a control group) and BTZ/Fe^{2+}@BTF/ALD, the real-time *in vivo* NIR-II fluorescence imaging was performed at different post-injection times by using an in vivo NIR-II fluorescence imaging system. The analysis of the signal intensity of NIR-II image was performed using the NIR-II *in vivo* imaging system software.

***In vivo* NIR-II Photothermal/Chemotherapy/Chemodynamic therapy.** When the tumor volume reached 90-120 mm^3, the 4T1 tumor-bearing mice or bone metastasis models mice were weighed, randomly divided into different treatment groups. About 12 h after the intravenous injection, the mice were divided into groups according to the corresponding treatment methods. Tumor volume and mice weight were assessed every other day in the next 14 days. After 14 days, these mice were sacrificed, and the tumors and their major organs were stained with hematoxylin-eosin (H&E) staining. The histological tumor sections were observed using an optical microscope. The weight of the tumors were also measured.

Statistical Analysis. The size distribution of NPs in TEM images were analyzed by Nano Measurer Software. Unless otherwise stated, statistical comparisons between various groups were performed using with one-way analysis of variance (ANOVA) with a corrected p value below 0.05 considered statistically to be significant (*$p < 0.05$, **$p < 0.01$, ***$p <0.001$). All data were generated from the mean values of three independent experiments and are presented as the mean ± standard deviation (s.d.).

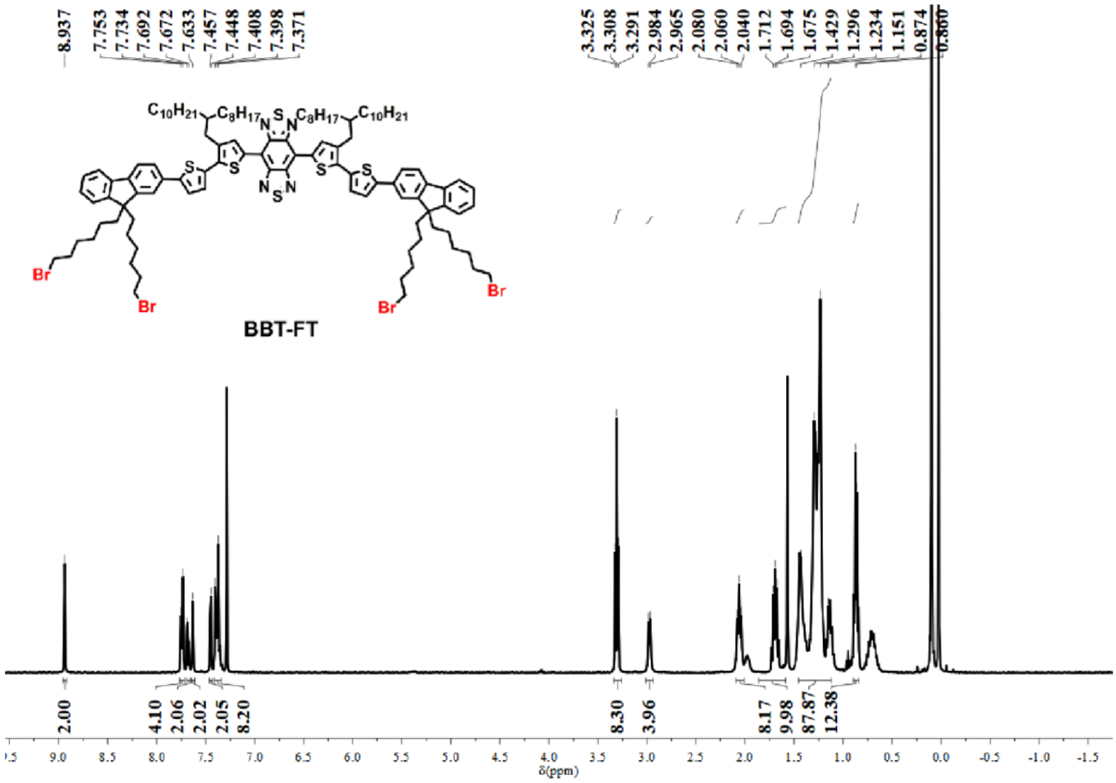

Figure S1. ^{1}H NMR spectrum of BBT-FT in $CDCl_3$.

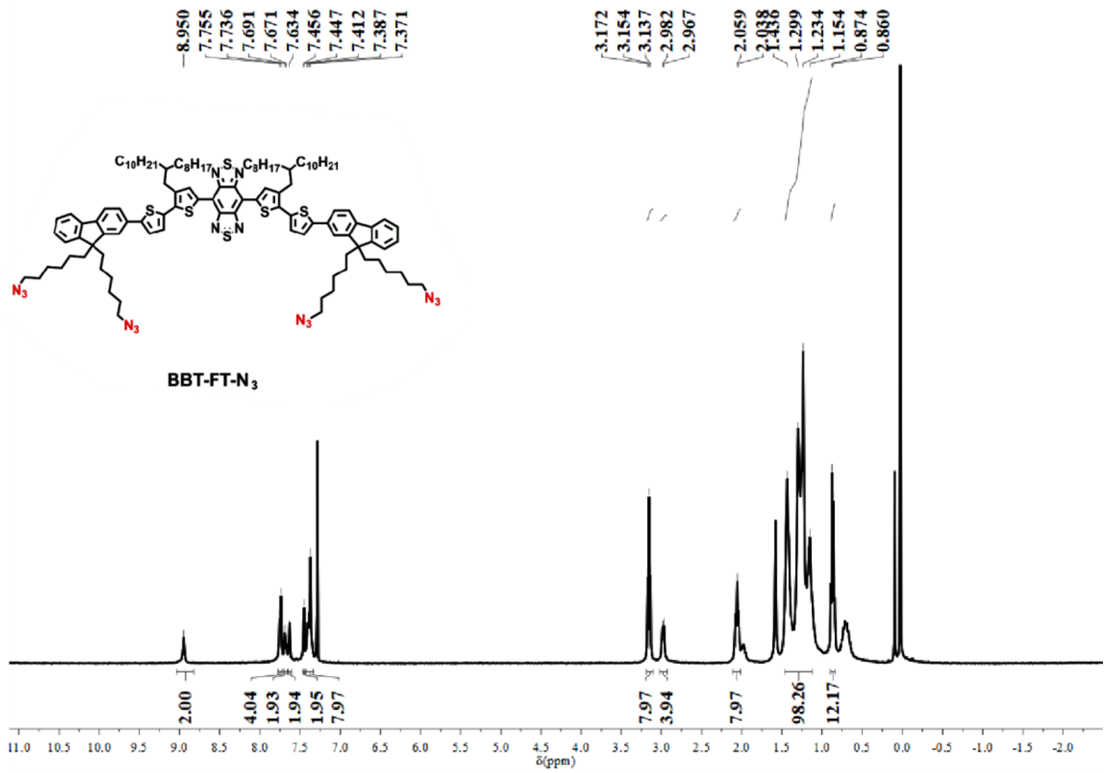

Figure S2. ^{1}H NMR spectrum of BBT-FT-N_3 in $CDCl_3$.

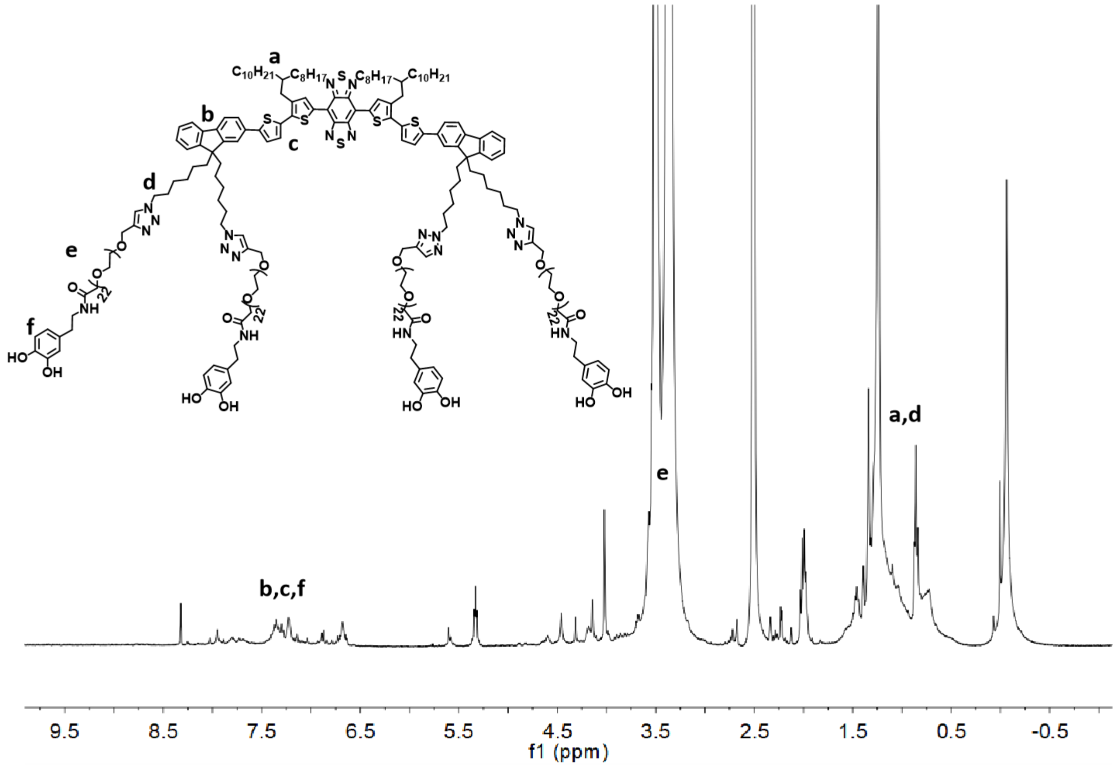

Figure S3. ^{1}H NMR spectrum of BBT-FT-DA in DMSO.

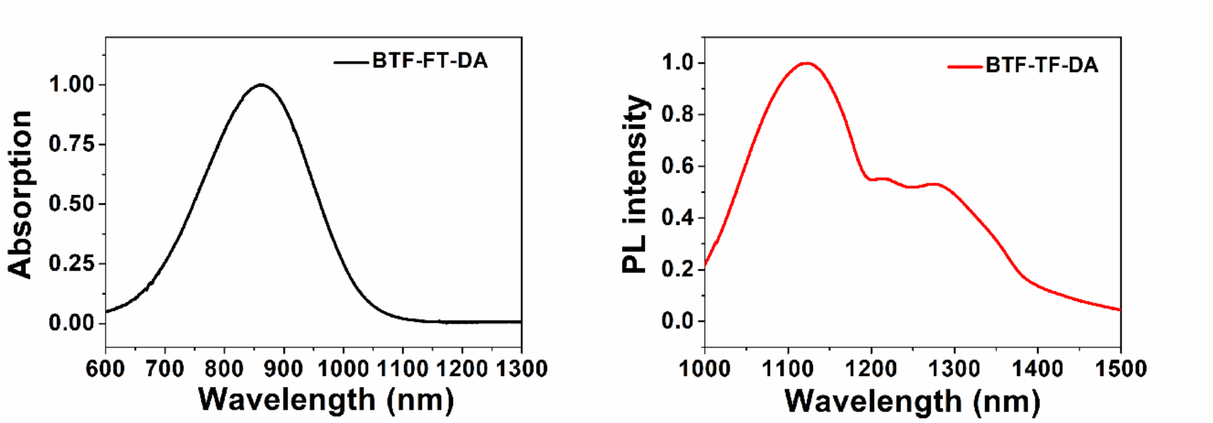

Figure S4. (A) Absorption and (B) fluorescence emission spectra of BBT-FT-DA (0.1 mg/mL) in THF.

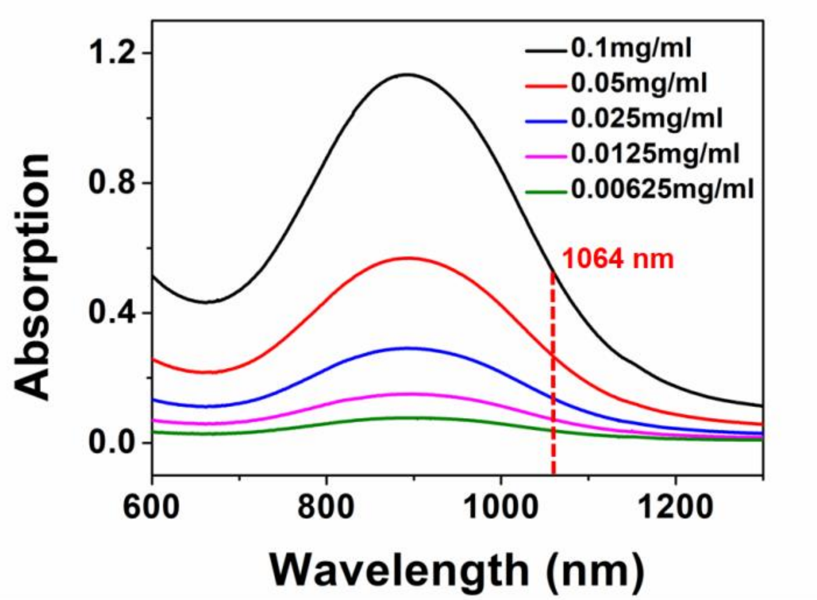

Figure S5. Absorption spectra of BTZ/Fe^{2+}@BTF-DA in water.

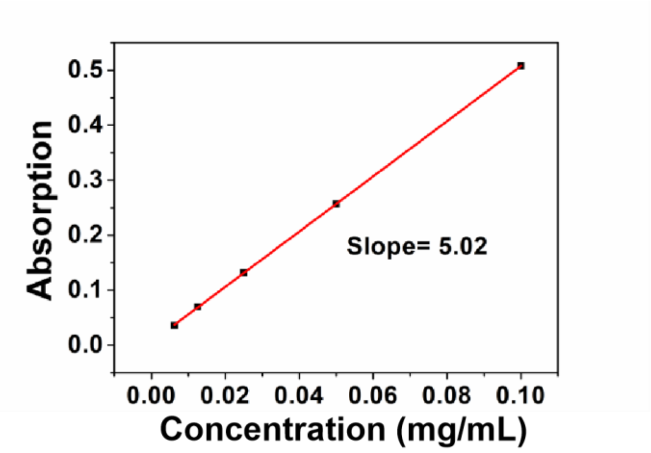

Figure S6. The mole extinction coefficient of BTZ/Fe^{2+}@BTF-DA at 1064 nm absorption.

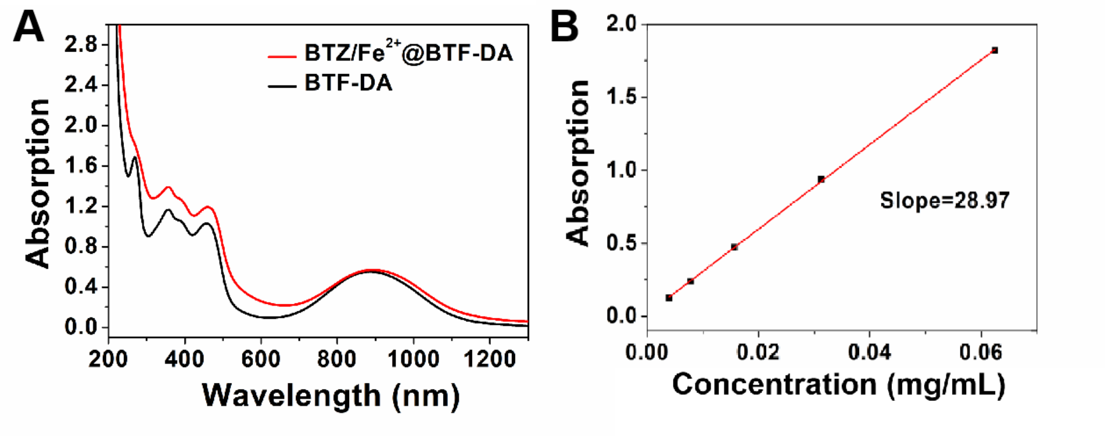

Figure S7. (A) Absorption spectra of BTZ/Fe^{2+}@BTF-DA in water (0.5 mg mL^{-1}). (B) Standard curve of BTZ at 270 nm.

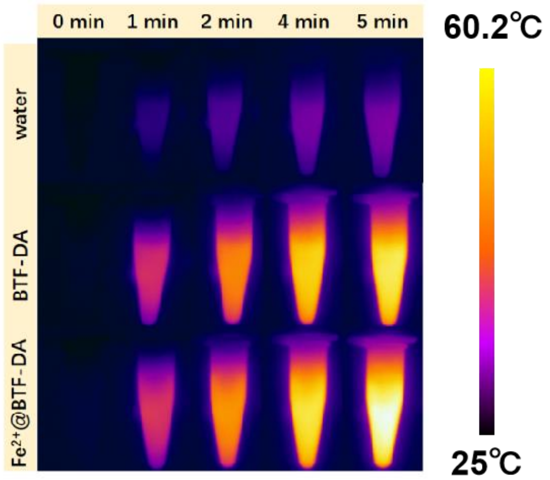

Figure S8. Photothermal experiment real-time imaging of water, BTF-DA and BTZ/Fe^{2+}@BTF-DA.

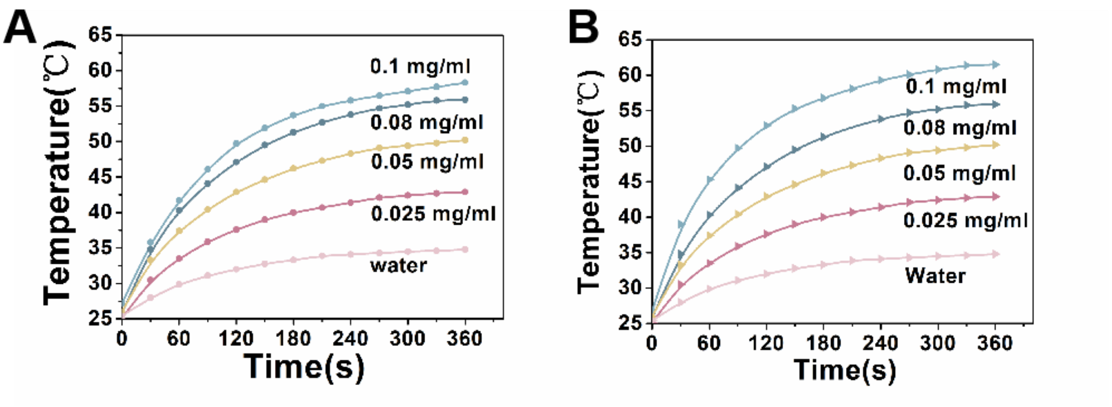

Figure S9. Photothermal heating curves of BTF-DA and BTZ/Fe^{2+}@BTF-DA at different concentrations under 1064 nm (1.0 W cm^{-2}) laser irradiation

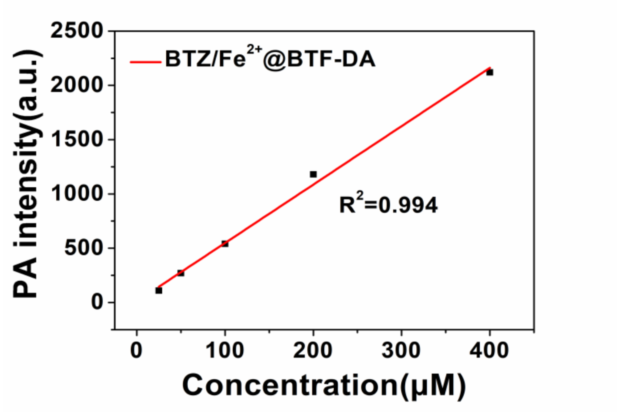

Figure S10. A linear relationship between PA signal and BTZ/Fe^{2+}@BTF-DA concentration.

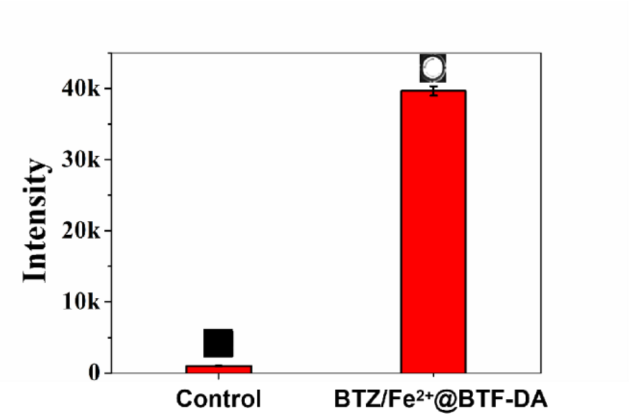

Figure S11. NIR-II fluorescence intensity of 4T1 cells incubated with or without BTZ/Fe^{2+}@BTF-DA. Insert: NIR-II fluorescence images of 4T1 cells incubated with or without BTZ/Fe^{2+}@BTF-DA.

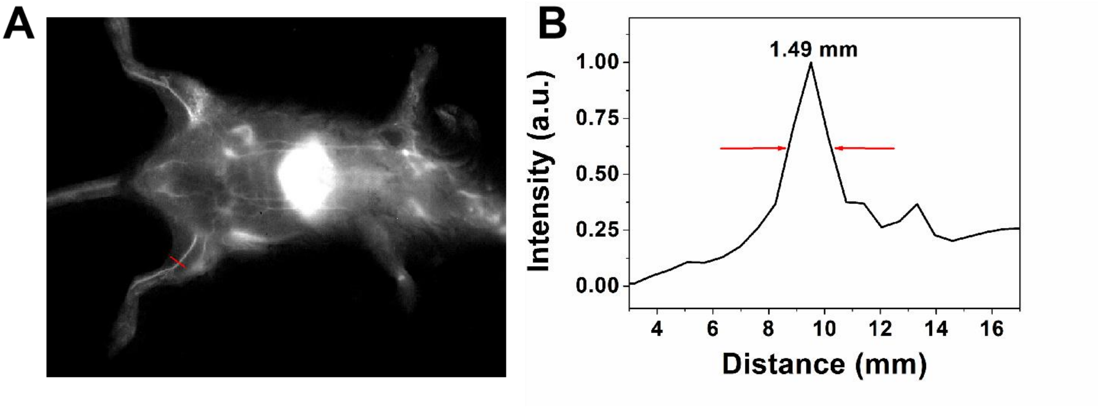

Figure S12. (A) NIR-II fluorescent angiography of mice under 1064nm laser. (B) The half-peak width at the blood vessel was selected.

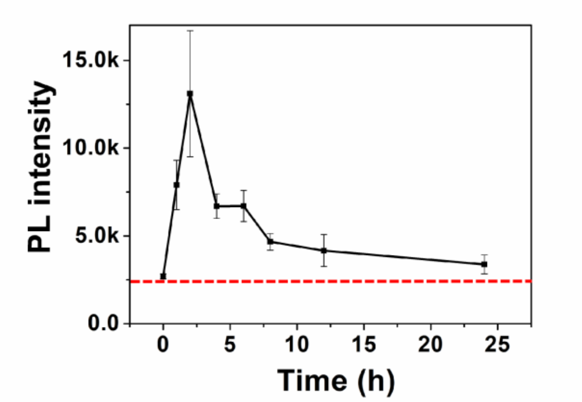

Figure S13. Blood circulation time curves of BTZ/Fe^{2+}@BTF/ALD administrated mice at various time points at 24 h post-injection. The red dashed line shows the fluorescence intensity of blood from mice without any treatment (control).

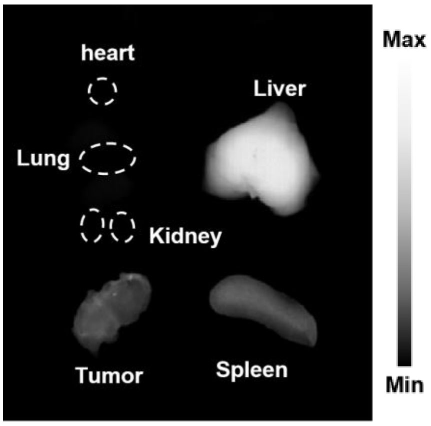

Figure S14. NIR-II fluorescence images of tumors and viscera of mice with 4T1 tumors after dissection.

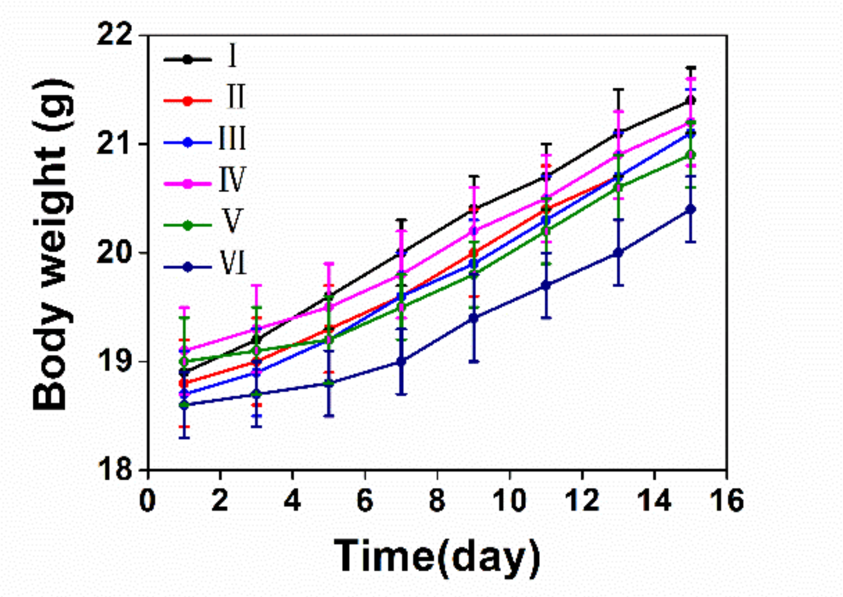

Figure S15. Body weight of mice with 4T1 tumor under different treatment (I: PBS, II: BTF-DA, III: BTZ@BTF-DA, IV: Fe^{2+}@BTF-DA, V: BTF-DA+1064 nm Laser, VI: BTZ/Fe^{2+}@BTF-DA+1064 nm Laser, 1.0 W cm^{-2}).

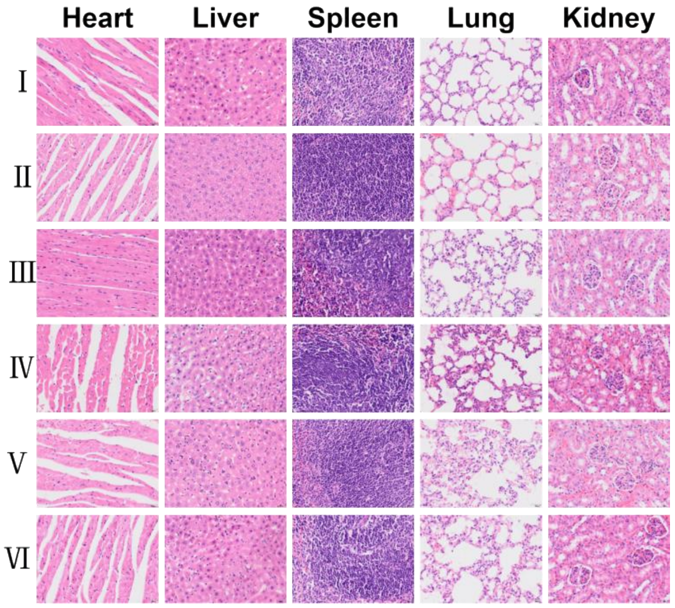

Figure S16. H&E staining images of major organs in 4T1 tumor mice (I: PBS, II: BTF-DA, III: BTZ@BTF-DA, IV: Fe^{2+}@BTF-DA, V: BTF-DA+1064 nm Laser, VI: BTZ/Fe^{2+}@BTF-DA+1064 nm Laser, 1.0 W cm^{-2}).

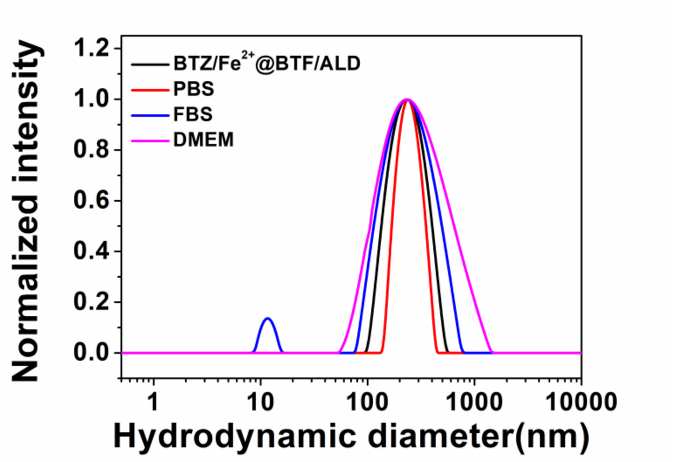

Figure S17. Particle size of BTZ/Fe^{2+}@BTF/ALD in aqueous solution, PBS, FBS and DMEM.

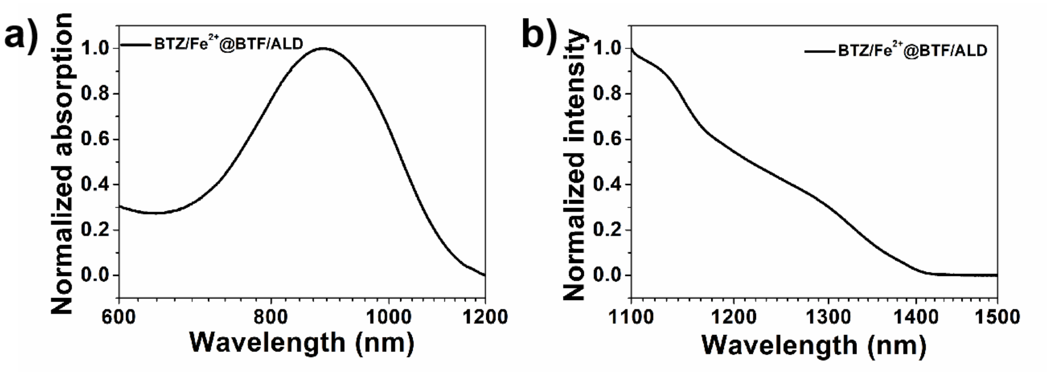

Figure S18. (a) Absorption and (b) fluorescence emission spectra of BTZ/Fe^{2+}@BTF/ALD (0.1 mg/mL) in aqueous solution

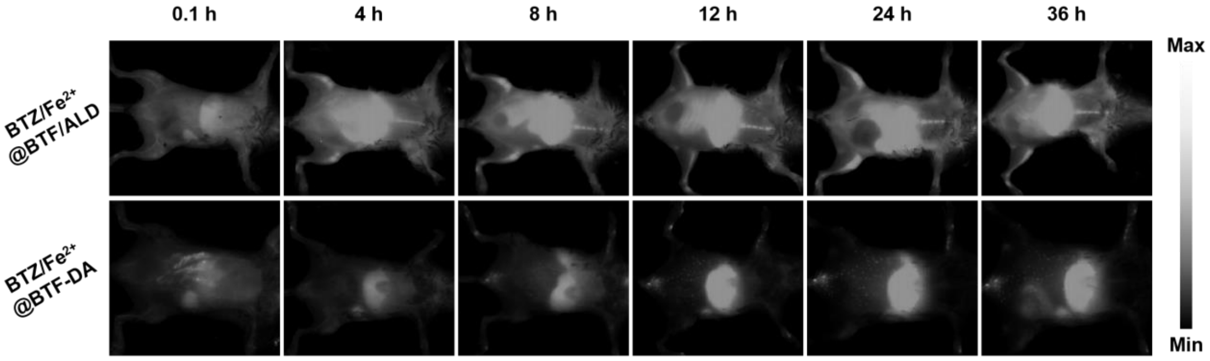

Figure S19. Bone targeted fluorescence imaging at different time intervals.

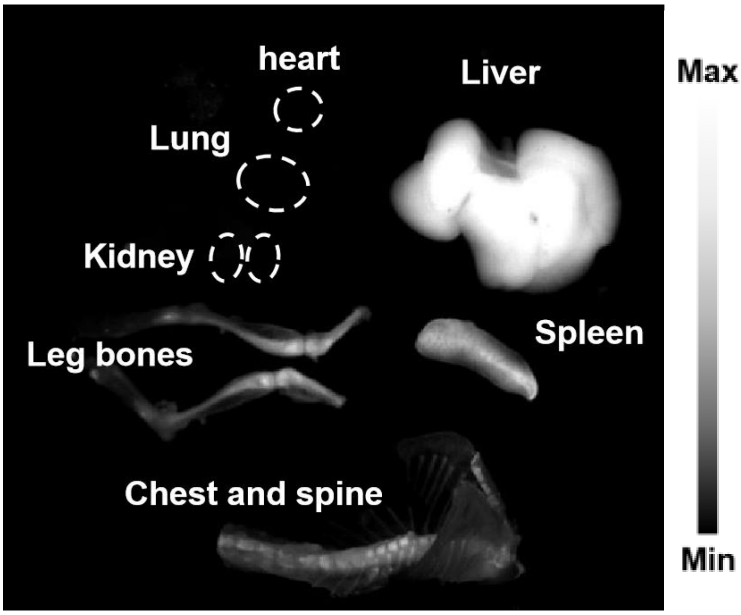

Figure S20. NIR-II fluorescence image of isolated organs and bones from normal mice injected with BTZ/Fe^{2+}@BTF/ALD.

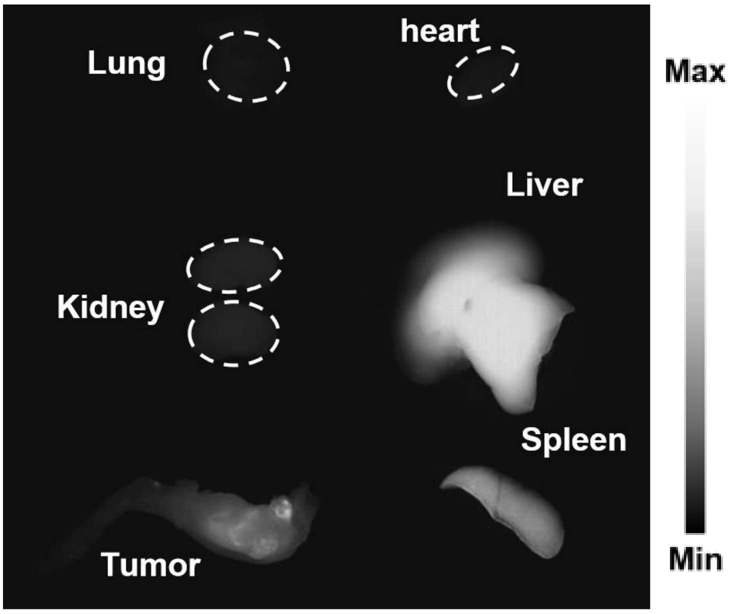

Figure S21. NIR-II fluorescence images of tumor and visceral NIR-II after dissection in a mouse model with bone metastases.

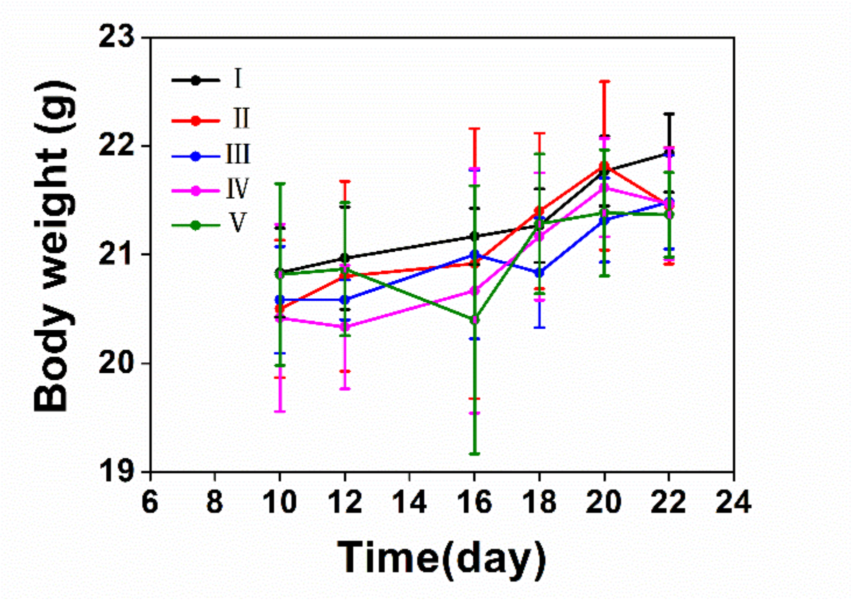

Figure S22. Body weight of mice with bone metastasis model under different treatment (I: PBS, II: BTF-DA, III: BTZ@BTF-DA, IV: Fe^{2+}@BTF-DA, V: BTF-DA+1064 nm Laser, VI: BTZ/Fe^{2+}@BTF-DA+1064 nm Laser, 1.0 W cm^{-2}).

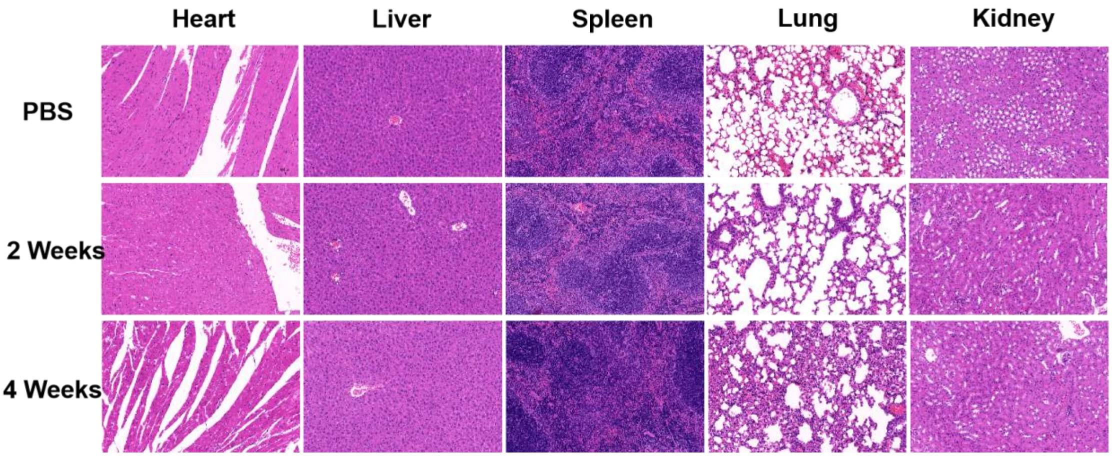

Figure S23. Image of major organs stained with H&E in a mouse model of bone metastases under treatment of BTZ/Fe^{2+}@BTF-DA+1064 nm Laser.

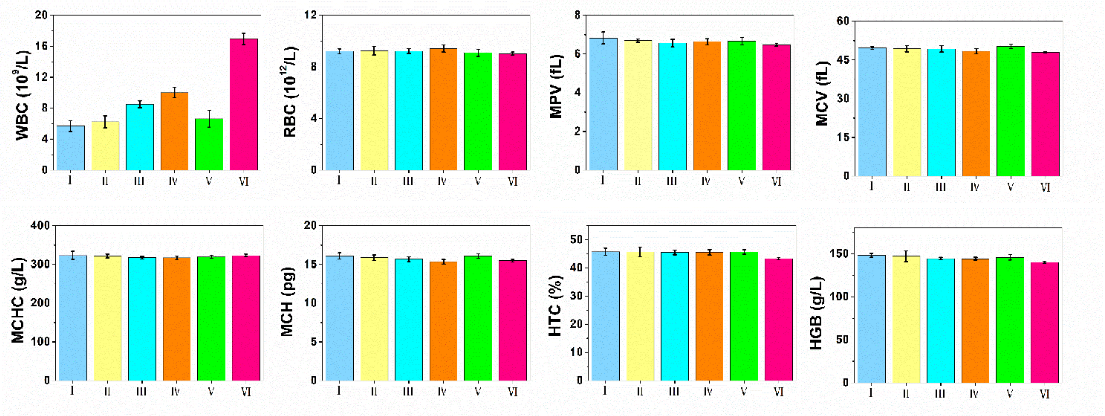

Figure S24. Blood analysis of the mice (I: PBS, II: BTF-DA, III: BTZ@BTF-DA, IV: Fe^{2+}@BTF-DA, V: BTF-DA+1064 nm Laser, Ⅵ: BTZ/Fe^{2+}@BTF-DA+1064 nm Laser, 1.0 W cm^{-2}).

References

[1] K. He, S. Chen, Y. Chen, J. Li, P. Sun, X. Lu, Q. Fan, W. Huang, *ACS Appl. Polym. Mater.* **2021**, *3*, 3238.
